# Natural and Synthetic AMPs from Latin America across Diversity Engineering and Applications

**DOI:** 10.1021/acsinfecdis.6c00394

**Published:** 2026-07-27

**Authors:** José Brango-Vanegas, Elizabete de Souza Cândido, Juliana Carneiro, Nanci Almeida Ribeiro, Octávio Luiz Franco

**Affiliations:** † Centro de Análises Proteômicas e Bioquímicas, Programa de Pós-Graduação em Ciências Genômicas e Biotecnologia, Universidade Católica de Brasília, Brasília-DF 71966-700, Brazil; ‡ S-inova Biotech, Programa de Pós-Graduação em Biotecnologia, 28106 Universidade Católica Dom Bosco, Campo Grande-MS 79117-900, Brazil

**Keywords:** Antimicrobial peptides, Latin America, Anti-infective, Engineering of AMPs, Antimicrobial resistance, Natural products, Drug discovery, Structure−activity relationship

## Abstract

Latin America’s biodiversity accounts for approximately 60% of the world’s total, fueling scientific research into natural products, a highly relevant area in drug discovery to this day. This is accompanied by an increasing number of antimicrobial peptides (AMPs) derived from natural products in the region, which over the past decade have been redesigned through modifications, enhancements, and refinements of their physicochemical properties and antimicrobial potency, using approaches that range from rational design and structure–activity relationship (SAR) studies to artificial intelligence and machine learning methodologies. AMPs and their derivatives represent a vast chemical space with potential for antibacterial, antifungal, antiviral, and antiparasitic therapies, with emerging applications beyond infectious diseases. Over the years, studies have identified these peptides from a wide range of sources, including amphibians, reptiles, invertebrates, and microorganisms from South America, highlighting their diverse biological activities. This raises important questions about whether Latin America is a fertile source of inspiration for the development of AMPs. It also prompts an examination of the region’s historical and ongoing contributions to the field of natural and synthetic AMPs with anti-infective potential. Additionally, it highlights the need to assess the region’s progress in keeping pace with the recent advancements in engineering AMPs to combat resistant bacteria. This mini-review addresses these topics and related issues, exploring Latin America’s discoveries, challenges, and innovations in AMPs and their applications, particularly in anti-infective agents.

## Graphical abstract

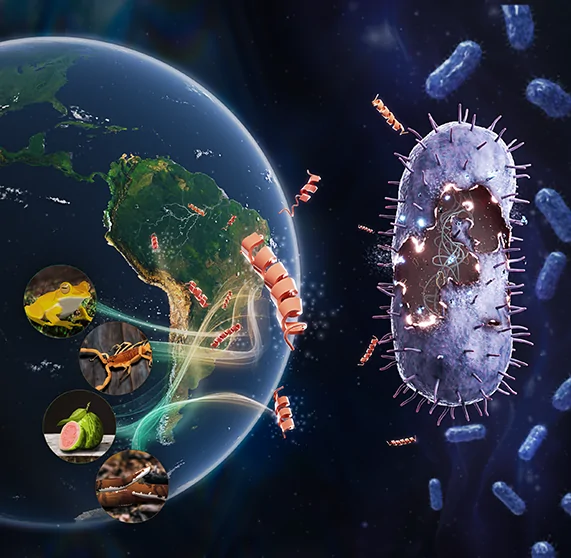

## 1 Introduction

Antimicrobial resistance (AMR) is currently recognized as one of the greatest global health crises of the 21st century, undermining the efficacy of conventional antibiotics and requiring the development of new anti-infective strategies.^1^ Recent estimates from the Global Burden of Disease (GBD) 2021 Antimicrobial Resistance Collaborators indicate that bacterial AMR was directly responsible for approximately 1.14 million deaths and was associated with 4.71 million deaths worldwide in 2021. Forecasts suggest that annual deaths directly attributable to AMR may reach 1.91 million by 2050, while AMR-associated deaths may exceed 8.22 million worldwide, underscoring the urgent need for novel antimicrobial interventions.^2^ Current surveillance data from the World Health Organization further demonstrate the continued global expansion of antibiotic resistance among clinically relevant bacterial pathogens, reinforcing AMR as a persistent and escalating public health challenge.^3^ Collectively, these findings reinforce the need for antimicrobial strategies capable of overcoming the limitations of conventional antibiotics.

This challenge posed by AMR is further exacerbated by biofilm formation, a microbial survival strategy associated with persistent microbial infections that can substantially increase the tolerance to antimicrobial agents. Biofilm-associated microorganisms exhibit intrinsic tolerance mechanisms involving extracellular matrix production, metabolic heterogeneity, persister cell formation, and enhanced horizontal gene transfer, which collectively contribute to antimicrobial tolerance, persistence, and treatment failure.^4^ As a result, biofilm-associated infections remain particularly difficult to eradicate and contribute significantly to recurrent infections, chronic disease progression, and healthcare-associated morbidity. These infections are frequently associated with chronic wounds, respiratory tract infections, and implanted medical devices, where biofilms act as reservoirs of multidrug-resistant microorganisms.^4^

Accumulating evidence has highlighted not only the growing complexity of biofilm-associated antimicrobial resistance but also significant geographical disparities in research focus and translational development. Countries in Asia, Latin America, and other biodiversity-rich regions tend to emphasize natural products, plant-derived antimicrobials, and antibiofilm strategies, whereas highly cited studies from North America and Europe are more frequently focused on clinical microbiology, pathogen genomics, resistance mechanisms, and translational therapeutic development.^5^ These regional differences partly reflect the unique biodiversity resources available in different parts of the world, particularly in Latin America, where natural products have become a major focus of antimicrobial research. Growing interest in biodiversity-driven drug discovery has stimulated the exploration of natural products, phytochemicals, and other bioactive compounds as promising sources of novel anti-infective agents.

Antimicrobial peptides (AMPs), typically ranging from 6 to 60 amino acids, characterized by their amphipathic nature and positive charge, have emerged as attractive candidates due to their broad-spectrum antimicrobial activity, diverse mechanisms of action, lower propensity for resistance development,^6,7^ and demonstrated ability to inhibit or disrupt microbial biofilms,^8,9^ particularly those formed by multidrug-resistant pathogens.^10,11^ Bibliometric analyses further indicate a rapid expansion of AMP-related research worldwide, particularly in studies targeting multidrug-resistant and biofilm-associated infections.^12^ Despite these advances, a substantial gap remains between biodiversity-driven antimicrobial discovery and clinical translation, particularly in regions such as Latin America, where many promising bioactive molecules remain insufficiently characterized with regard to their mechanisms of action, structural properties, scalability, and therapeutic potential.

In this context, AMPs have attracted considerable attention because of their multifunctional properties and remarkable structural diversity.This diversity has created opportunities for rational peptide engineering, enabling the optimization of physicochemical properties, antimicrobial potency, stability, and selectivity^13^ through rational engineering strategies supported by computational and experimental methodologies. AMPs exhibit immunomodulatory and adjuvant properties,^6,14^ which makes them attractive targets for bioprospecting and pharmacological engineering. Biodiversity-rich ecosystems, therefore, represent valuable reservoirs of molecular diversity for the discovery of novel peptide scaffolds. Latin America, one of the most biodiverse regions in the world, offers access to a vast repertoire of natural scaffolds that can serve as starting points for peptide discovery and subsequent optimization.

Modern computational techniques, including peptide modeling, molecular docking, machine learning (ML), and artificial intelligence (AI), have transformed AMP discovery and optimization. These approaches enable the efficient exploration of the vast peptide sequence space. Advances in deep learning have enabled frameworks capable of extracting statistical patterns from large peptide databases to generate new sequences with balanced physicochemical and biological properties, significantly reducing the number of candidates required for experimental testing.^15,16^

While computational approaches have accelerated the discovery of AMPs, experimental studies based on natural products remain indispensable. Accordingly, studies on extracts and compounds isolated from plants, microorganisms, and neotropical animals across the region demonstrate significant antimicrobial activity against pathogenic bacteria and fungi, including resistant strains, underscoring the value of Latin American biodiversity for antimicrobial discovery.^17–21^ Peptides and secretions from insects, amphibians, and aquatic organisms have been widely described as sources of antimicrobial molecules and structural templates for the design of synthetic peptides.^22–25^ For instance, the exceptional amphibian diversity of tropical forests^22^ and the presence of AMP families, such as antilipopolysaccharide factors (ALFs), in crustaceans such as the white shrimp *Litopenaeus vannamei* illustrate the breadth of innate defense molecules available for pharmacological exploration.^25^ Although these studies collectively reveal the remarkable diversity of AMPs across Latin American taxa, the available knowledge remains scattered among different organisms, molecular classes, and research disciplines, limiting a comprehensive understanding of their structural diversity, engineering potential, and translational applications.

Despite the remarkable diversity of AMPs identified across Latin American biodiversity, their characterization and application remains with few comprehensive reviews integrating molecular classes, biochemical properties, engineering strategies, and biotechnological applications.^26,27^ This fragmented landscape has also limited the translation of promising discoveries into intellectual property and clinically relevant products. For instance, the Neotropical region accounts for only 3.7% of frog-derived AMP patents, underscoring the need for greater integration among basic research, peptide engineering, and drug development.^28^

Importantly, AMP biological activity is intrinsically influenced by their three-dimensional conformation, stereochemistry, amphipathicity, and molecular stability, all of which contribute to membrane interactions, biological selectivity, proteolytic resistance, and antimicrobial efficacy.^29–32^ Architectural elements such as α-helical folding, β-sheet organization stabilized by disulfide bonds, cyclization, and other constrained topologies represent some of the principal molecular architectures underlying these functional properties.^29–31,33^ Furthermore, stereochemical modifications, including the incorporation of *D*-amino acids and other configurational variations, can substantially influence peptide stability, biological selectivity, and antimicrobial efficacy, underscoring the importance of stereochemistry in AMP design and optimization.^32^ To characterize these molecular properties, characterization approaches including nuclear magnetic resonance (NMR), mass spectrometry (MS/MS), circular dichroism (CD), and advanced computational modeling have become essential tools for elucidating structure–activity relationships (SAR) and validating stereochemical assignments. Together, these approaches provide critical insights into AMP function, enabling the identification of SAR that guide peptide engineering and optimization strategies.^34,35^

Considering these challenges, this Mini-Review explores the biological richness of Latin America through the lens of modern rational design and conjugation strategies. It consolidates data on natural and synthetic AMPs, identifies regional knowledge gaps, and outlines research priorities that may facilitate the development of these peptides into clinical and biotechnological applications.

## 2 Natural Sources of AMPs in Latin America

The Latin American region is recognized for its extraordinary biodiversity, encompassing a vast diversity of animals, plants, and microbes. This biological richness represents a valuable reservoir of natural AMPs, many of which have remained unexplored. Over the past few decades, research groups across the region have contributed significantly to the identification and characterization of these molecules, paving the way for the development of novel anti-infective strategies inspired by natural compounds. In this context, AMPs derived from microbial, plant, and animal sources, including those from marine and terrestrial environments, are discussed below.

### 2.1 Microbial Sources of AMPs Terrestrial and Marine Environments

The most prominent and consistently active Latin American groups working on the discovery and molecular characterization of microbial antimicrobial peptides are primarily based in Argentina, Brazil, and Mexico. Their most notable research focuses on bacteriocins,^36–39^ microcins,^40^ nonribosomally synthesized peptides, including peptaibols from endophytic fungi,^41^ and by marine bacteria, as well as lipopeptides from terrestrial bacteria (Table 1).

**Table 1:** Antimicrobial Peptides from Microorganism from Latin American Described or Characterized in Primary Studies

Peptide name	Family/Class	^a^Molecular mass	Sequence	^b^MIC range	Specie	Ref.
Lactocin 70^c^	Bacteriocin	∼3500	GMSGYIQGIPDFLKGYLHGISAANKHKKGRL	NR	*Lactobacillus casei*	^36^
Hyicin 3682	Bacteriocin	2117	ITSFSLCTPGCAKTGSFNSYCC	NR	*Staphylococcus hyicus* 3682	^37^
Hyicin 4242	Bacteriocin	∼3100	NKGCSACAIGAACLADGPIPDFEVAGITGTGIAS	NR	*Staphylococcus hyicus* 4242	^38^
Microcin J25	Class II lasso peptide	∼2100	GGAGHVPEYFVGIGTPISFYG	0.004–2.3	*Escherichia coli*	^40^
Pelgipeptin A	NRPs - Lipopeptide	1073	C6-Dab-V-Dab-FL-Dab-VLS	7.5–59.6	*Paenibacillus elgii* AC13	^51^
Pelgipeptin B	NRPs - Lipopeptide	1101	C7-Dab-I-Dab-FL-Dab-VLS	3.6–7.3	*Paenibacillus elgii* AC13	^51^
Pelgipeptin C	NRPs - Lipopeptide	1087	C7-Dab-V-Dab-FL-Dab- VLS	7.4–58.8	*Paenibacillus elgii* AC13	^51^
Pelgipeptin D	NRPs - Lipopeptide	1087	C6-Dab-I-Dab-FL-Dab- VLS	7.4–29.4	*Paenibacillus elgii* AC13	^51^
L2	NRPs	501.3	_D_A_D_V_D_AW-Orn-T-Orn-_D_VK-NH_2_	>1000	*Streptomyces* sp. H-KF8	^52^
L3	NRPs	698.4	W_D_A_D_V_D_AW-Orn-T-Orn-_D_V-Y(NO_2_)-K-NH_2_	90–350	*Streptomyces* sp. H-KF8	^52^
Aureocin 53	*N*-formylated antimicrobial peptide	∼6012	MSWLNFLKYIAKYGKKAVSAAWKYKGKVLEWLNVGPTLEWVWQKLKKIAGL	NR	*Staphylococcus aureus* A53	^39,46^
Trilongin BI	NRPs - Peptaibols	1935	Ac-Aib-A-Aib-A-Aib-AQ-Aib-Vxx-Aib-G-Lxx-Aib-P-Vxx-Aib-Aib-QQ-Pheol	40	*Trichoderma* sp. P8BDA1F1	^41,53^
Trilongin BII	NRPs - Peptaibols	1949	Ac-Aib-A-Aib-A-Aib-AQ-Aib-Vxx-Aib-G-Lxx-Aib-P-Vxx-Aib-Vxx-QQ-Pheol	320	*Trichoderma* sp. P8BDA1F1	^41,53^
Trilongin BIII	NRPs - Peptaibols	1949	Ac-Aib-A-Aib-A-Aib-Aib-Q-Aib-Vxx-Aib-G-Lxx-Aib-P-Vxx-Aib-Aib-QQ-Pheol	160	*Trichoderma* sp. P8BDA1F1	^41,53^
Trilongin BIV	NRPs - Peptaibols	1963	Ac-Aib-A-Aib-A-Aib-Aib-Q-Aib-Vxx-Aib-G-Lxx-Aib-P-Vxx-Aib-Vxx-QQ-Pheol	310	*Trichoderma* sp. P8BDA1F1	^41,53^

a Molecular mass in Da corresponds to the mature bioactive peptide, reported or calculated for the modified form when explicitly stated.

b MIC ranges in μM summarize antimicrobial activity reported against bacteria and/or fungi using broth microdilution or equivalent assays.

c The reported sequence corresponds to the *N*-terminal region, and the molecular mass does not correspond to this sequence but rather to that of the peptide-enriched fraction visualized by gel electrophoresis. NR, not reported. Dab is 2,4-diaminobutyric acid. Orn is ornithine. Y­(NO_2_) is nitrotyrosine. Aib is α-aminoisobutyric acid. Vxx is valaline or isovaline. Lxx is leucine or isoleucine. Pheol is phenylalaninol.

Specifically, the bacteriocin lactocin 705, produced by *Lactobacillus casei* CRL 705 isolated from dry fermented sausage, was identified in Argentina. Extracts enriched in this peptide exhibited antimicrobial activity against *Lactobacillus plantarum* CRL 69 in antibiogram-type assays.^36^ Furthermore, Argentine research groups have extensively investigated bacteriocins produced by lactic acid bacteria, including enterocins and lactocins, focusing on their antimicrobial activity, biochemical properties, and applications in food biopreservation, as well as advancing the understanding of their genetic diversity and biosynthetic mechanisms.^42,43^ One example is the characterization of enterocin CRL35, a bacteriocin produced by the strain *Enterococcus faecium* CRL35, isolated from artisanal cheeses from Argentina. This proteinaceous peptide is notable for its high stability, retaining biological activity under extreme pH conditions (2–10) and after heat treatment (100 °C for 30 min). Enterocin CRL35 exhibits potent inhibitory activity against foodborne pathogens, being particularly effective against various strains of *Listeria monocytogenes* and *Staphylococcus aureus*. These findings suggest that both the *E. faecium* CRL35 strain and its purified bacteriocin represent promising biopreservatives for the food industry, offering a natural alternative for controlling *Listeria* contamination in dairy products.^44^

Pioneering studies in Argentina identified microcin J25 (Figure 1B), a lasso peptide produced by *Escherichia coli*, which was shown to be active against clinically derived strains of *E. coli*, *Salmonella*, and *Shigella* in antibiogram-type assays using 1.5 μg. The MICs ranged from 0.004 to 2.3 μM across different *E. coli* strains, and activity was also observed against a single strain of *Salmonella Newport*.^40^ Subsequently, microcin J25 was comprehensively characterized by MS/MS and NMR analyses, revealing that it is not a simple cyclic peptide but a unique 21-residue lariat protoknot (class II lasso peptide), in which a linear *C*-terminal tail threads through an *N*-terminal macrolactam ring. This stereochemically constrained lasso topology defines its three-dimensional organization and contributes directly to the remarkable thermal stability, proteolytic resistance, and biological activity of microcin J25.^45^

**Figure 1:**
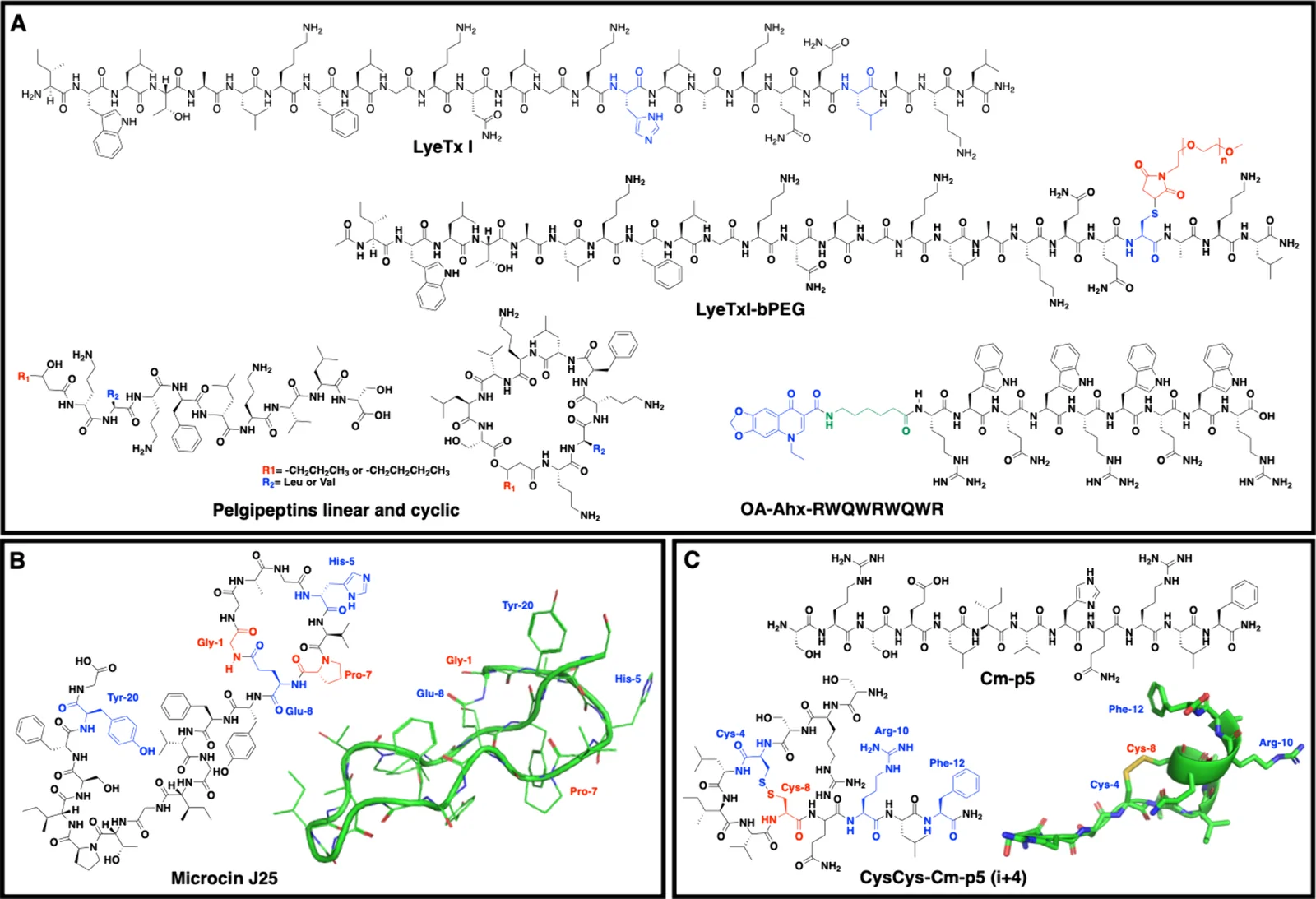
Representative chemical structures of AMPs and engineered AMPs developed in Latin America. (A) Chemical structures of selected AMPs and AMP derivatives. Pelgipeptins comprise a family of five canonical cyclic homologues (pelgipeptins A–E). The producing strain, *Paenibacillus elgii* AC13, isolated in Brazil, simultaneously secretes both the canonical cyclic peptides and their corresponding linear counterparts. Also shown is LyeTxI-bPEG, a PEGylated analogue of LyeTxI, an AMP originally isolated from the Brazilian spider *Lycosa erythrognatha*. In addition, the figure includes OA-Ahx-RWQWRWQWR, a conjugated peptide derived from bovine lactoferricin B, in which the *N*-terminus is functionalized with the nonpeptidic moieties oxolinic acid (OA) and 6-aminohexanoic acid (Ahx). This peptide was developed in Colombia. (B) Chemical structure and three-dimensional structure of microcin J25 (PDB ID: 1PP5). Originally identified in Argentina from *Escherichia coli*, microcin J25 is a 21-residue lasso peptide (class II) characterized by a *C*-terminal tail threaded through an *N*-terminal macrolactam ring. The characteristic lasso architecture of microcin J25 underlies its exceptional thermal stability, resistance to proteolysis, and antimicrobial activity. (C) Chemical structure and computationally modeled three-dimensional structure of CysCys-*Cm*-p5, an *(i + 4)* disulfide-bridged derivative of *Cm*-p5, a bioinspired peptide designed from a native AMP identified in the marine snail *Cenchritis muricatus* from Cuba.

In parallel with these efforts in Argentina, research groups in Brazil have also made significant contributions. In Brazil, a series of bacteriocins identified by the same research group highlights a sustained effort to explore AMPs through integrated approaches. These include genomic analysis, heterologous expression, and mechanistic evaluation. The studies target multidrug-resistant pathogens, bovine mastitis-associated bacteria, and clinically relevant staphylococcal infections. Initially, hyicin 3682 was described for the strain *Staphylococcus hyicus* 3682, isolated from bovine milk, which was identified through genome sequencing as well as from enriched fractions obtained by protein precipitation of culture supernatants, which exhibited antimicrobial activity against *Micrococcus luteus* ATCC 4698 and *Staphylococcus aureus* 4S1. Notably, these findings indicate that hyicin 3682 exerts a bactericidal effect on both strains, as evidenced by a significant reduction in viable cell counts. However, only *M. luteus* ATCC 4698 cells were partially lysed by this bacteriocin.^37^ Subsequently, the same group identified hyicin 4244, a small antimicrobial peptide detected in the culture supernatant of *Staphylococcus hyicus* 4244. This peptide exhibits a relatively narrow antimicrobial spectrum, primarily targeting staphylococcal species associated with human infections (*S. saprophyticus* 46s) and bovine mastitis (*S. aureus* 4124), with activity levels of 256 and 128 AU·mL^–1^, both of which were effective against the tested strains. Notably, hyicin 4244 shows a pronounced ability to inhibit biofilm formation at a subinhibitory concentrations (128 AU·mL^–1^) in *S. saprophyticus* 46s, with antibiofilm activity being its most distinctive functional feature rather than strong planktonic bactericidal effects.^38^

Expanding this line of investigation, aureocin A53, a highly cationic 51-residue *N*-formylated antimicrobial peptide produced by *Staphylococcus aureus* A53, was also characterized as part of efforts to identify potent antimastitis agents.^46^ Recently, it has been demonstrated that this peptide exhibits a broad antimicrobial spectrum, effectively inhibiting the growth of multiple staphylococcal and streptococcal strains isolated from bovine mastitis, including multidrug-resistant isolates, with activity levels reaching up to 2048 AU·mL^–1^. Notably, it demonstrates bactericidal activity and a lytic mode of action against susceptible strains, shows no cytotoxic effects on bovine mammary epithelial cells after 24 h of exposure, and retains activity in *ex vivo* models.^39^ Collectively, these studies illustrate a coherent research trajectory, progressing from peptide identification and characterization toward the development of functionally diverse antimicrobial candidates with potential applications in the control of bovine mastitis.

In addition to staphylococcal bacteriocins, Bacillus species have also been extensively explored as sources of structurally diverse antimicrobial peptides. A study conducted between Peru and Brazil investigated a strain of *Bacillus velezensis* TCSH0001, isolated from *tocosh* (a traditional Peruvian fermented potato product) as a source of AMPs through genome mining. Whole-genome sequencing revealed multiple biosynthetic gene clusters associated with compounds exhibiting strong antimicrobial activity, including nonribosomal lipopeptides such as surfactin, fengycin, and bacillomycin D as well as ribosomally synthesized and post-translationally modified peptides (RiPPs), notably plantazolicin. The dipeptide bacilysin was also identified and can be considered a simple AMP because of its antimicrobial properties. Functional analyses by RT-qPCR confirmed the expression of key biosynthetic genes, including *bmyA*, associated with bacillomycin D production during the bacterial growth. Complementary in vitro antagonism assays demonstrated strong inhibitory activity against *Staphylococcus aureus* (inhibition zones up to 14 mm) and moderate activity against *Escherichia coli* (3 mm).^47^

Pelgipeptins represent an interesting group of cyclic lipopeptides primarily produced by species of the genus *Paenibacillus*. They belong to the class of nonribosomal peptide (NRP) metabolites and have gained attention as promising anti-infective molecules. The *P. elgii* AC13 strain, isolated from soil samples collected in the Brazilian Cerrado biome, demonstrated high biotechnological potential due to its remarkable antimicrobial activity. Through genomic sequencing and subsequent bioinformatic analysis, the presence of sequences encoding 40 nonribosomal peptide synthetase (NRPS) modules was revealed. Thus, a gene cluster responsible for pelgipeptin biosynthesis was identified, along with other genes associated with fusaricidin and bacteriocin production.^48^ These cyclic lipopeptides, with the general sequence (Dab-X-Dab-FL-Dab-VLS-COOH, where X can be L or V), contain a hydrophobic fatty acid moiety (C6–C7) attached to the *N*-terminus of the first residue, 2,4-diaminobutyric acid (Dab). This acyl chain also promotes cyclization through the formation of an ester bond between its C3 hydroxyl group and the *C*-terminal carboxyl group of the serine residue.^49^

The *P. elgii* AC13 strain was shown to produce antimicrobial lipopeptides displaying an unusual co-occurrence of cyclic and linear forms in liquid cultures. Although pelgipeptins are typically described as a family of five strictly cyclic homologues (A–E), this strain appears to simultaneously secrete both the canonical cyclic structures and their linear counterparts (Figure 1A). The presence of linear isoforms may be attributed either to reduced thioesterase activity, responsible for macrocyclization during biosynthesis, or to the action of unidentified proteases during bacterial growth. Importantly, control experiments demonstrated that purification and extraction procedures did not induce artificial hydrolysis, confirming that linearization is a genuine biological feature rather than a technical artifact. Functionally, MIC assays highlighted the critical role of cyclization in antimicrobial potency. Cyclic pelgipeptins (A–D) exhibited significantly lower MIC values (6.25–12.5 μM) against *E. coli* and *S. aureus*, whereas the linear counterparts of isoforms B and C showed markedly reduced activity, with MIC values exceeding 100 μM. Conversely, the linear forms exhibited lower cytotoxicity toward human fibroblasts than the cyclic molecules. The marked differences observed between cyclic and linear isoforms suggest that conformational restriction is a critical determinant of antimicrobial potency, membrane-targeting efficiency, and cellular toxicity.^50^ Pelgipeptin isoforms A, B, C, and D, as well as a fraction containing all isoforms, were also evaluated for their antifungal activity against a range of human-pathogenic fungi. The results showed that pelgipeptin B and the pelgipeptin mixture exhibited the highest efficacy, with MIC values of 4 μg·mL^–1^ (3.6 μM) against *Cryptococcus neoformans* H99, 8 g·mL^–1^ (7.3 M) against *Candida albicans* SC5314, and 8 g·mL^–1^ (7.3 M) against *Paracoccidioides brasiliensis* Pb18. For *Candida glabrata* ATCC 90030, the MIC value was 32 μg·mL^–1^ (29.1 μM). One of the most relevant highlights of the study was the ability of these lipopeptides to combat biofilms: while fluconazole showed no efficacy in inhibiting *C. albicans* biofilm formation, pelgipeptins exhibited an SMIC80 of 16 g·mL^–1^ and reduced cell viability both during the adhesion phase and in preformed biofilms (24h). Structurally, these molecules are cyclic cationic peptides that act on the fungal membrane, positioning them as viable alternatives for the treatment of systemic mycoses, especially in cases of resistance to conventional antifungals.^51^

In addition to terrestrial microorganisms, marine environments also represent a rich and increasingly explored source of AMPs. Marine microorganisms, particularly bacteria associated with seawater, sediments, and marine invertebrates, represent an important source of AMPs, including both ribosomally synthesized peptides and NRPs. These compounds are produced as part of microbial competition in complex environments and display diverse structures and mechanisms of action, often involving membrane disruption or interference with essential cellular processes. Within this global framework, increasing attention has been directed toward marine microorganisms from Latin American environments, as growing evidence indicates that isolates from this region also contribute significantly to this diversity. For instance, marine-derived *Streptomyces* sp. H-KF8 strains isolated from Chilean Patagonia have been shown, through genome mining and experimental validation, to produce NRPs with antimicrobial activity, including cyclic peptides with membrane-targeting mechanisms. Predictions from NRP biosynthetic pathways enabled the design of eight analogs (five linear and three cyclic) that incorporate *D*-amino acids, nitrotyrosine (Y­(NO_2_)), and ornithine (Orn) to enhance stability and cationic charge. Among the designed and synthesized peptides, the linear peptides L2 (_D_A_D_V_D_AW-Orn-T-Orn-_D_VK-NH_2_) and L3 (W_D_A_D_V_D_A-W-Orn-T-Orn-_D_V-Y­(NO_2_)-K-NH_2_), exhibited the highest potency. In biological assays against strains such as *Staphylococcus aureus*, *Escherichia coli*, and *Candida albicans*, peptide L3 demonstrated notable minimum microbicidal concentration (MMC_99_) values at 4, 6, and 26 h, ranging from 6.3 to 25 μg·mL^–1^ across all three microorganisms, with MIC values between 90 and 350 μM. Mechanistically, this peptide acts through direct interaction with the cell membrane, leading to dissipation of the transmembrane potential and nucleoid relaxation, without causing significant hemolysis or cytotoxicity in human cells (HEK and HepG2), positioning these molecules as promising candidates for the treatment of multidrug-resistant infections. Although membrane perturbation appears to be the primary mechanism, nucleoid relaxation and DNA packing defects suggest the possibility of secondary intracellular effects that remain to be fully elucidated.^52^

Peptaibols are linear, nonribosomally synthesized secondary metabolites characterized by a high content of the nonproteinogenic amino acid α-aminoisobutyric acid (Aib), as well as an acylated *N*-terminus and a *C*-terminal amino alcohol. This distinctive chemical composition enables these molecules to adopt stable helical conformations, allowing them to interact with biological membranes by forming ion channels or pores.^53^ Representative members of this class include tricholongins, longibrachins, trichobrachins, and trichovirins. Another group, the trilongins BI, BII, BIII, and BIV, which are peptaibols containing 20 amino acid residues, was identified from the endophytic fungus *Trichoderma* sp. P8BDA1F1, isolated from *Begonia venosa* collected in Brazil. These compounds exhibited notable antifungal activity against the phytopathogen *Colletotrichum gloeosporioides*, with MIC values of 40 μM for BI, 320 μM for BII, 160 μM for BIII, and 310 μM for BIV. In addition, these peptides demonstrated the ability to inhibit the proteasome, specifically targeting the chymotrypsin-like subunit, highlighting their dual biotechnological applicability. The ability of trilongins to form ion-conducting channels while also displaying proteasome inhibitory activity highlights the mechanistic complexity that can emerge among membrane-active peptide systems. Although the trilongins showed considerable cytotoxicity, limiting their selectivity in parasitic infection models such as leishmaniasis, this study represents the first to correlate these specific peptaibols with proteasome inhibition.^41^ The diversity of biological activities observed among these peptaibols illustrates the potential of endophytic fungi as sources of compounds for both biomedical and agricultural applications.

Importantly, advances in genome mining, peptide engineering, and functional assays have significantly expanded the discovery of novel antimicrobial peptides while enabling the rational optimization of their activities, stability, and selectivity. Collectively, examples from Latin American ecosystems highlight the vast yet underexplored biotechnological value of these regions. Microbial ecosystems, therefore, remain an important reservoir of bioactive molecules for the development of antimicrobial, agricultural, and biopreservative applications.

### 2.2 Plant Defense Structure and Antimicrobial Potential

While microbial sources have provided a wealth of antimicrobial compounds, plants also represent a complementary source of AMPs. Plant-derived AMPs from native Latin American flora comprise a structurally diverse and pharmacologically promising group of anti-infective molecules. The studies summarized in Table 2 were predominantly conducted in Brazil, underscoring the role of regionally biodiversity-driven research programs.

**Table 2:** Antimicrobial Peptides from Plants from Latin American Described or Characterized in Primary Studies

Peptide name	Family/Class	^a^Molecular mass	Sequence	^b^MIC range	Specie	Ref.
PvD1	Plant defensin	∼5320	KTCENLADTYKGPCFTTGSCDDHCKNKEHLRSGRCRDDFRCWCTKNC	4.7–18.8	*Phaseolus vulgaris*	^55^
PvD1r	Plant defensin	∼6000	MKTCENLADTYKGPCFTTGSCDDHCKNKEHLRSGRCRDDFRCWCT​KNC	∼16.6	*Phaseolus vulgaris*	^56^
Pg-AMP1	Glycine-rich AMP (GRP)	∼6029	RESPSSRMECYEQAERYGYGGYGGGRYGGGYGSGRGQPVGQGVERS​​HDD​NRNQPR	∼6.6	*Psidium guajava*	^57^
La-AMP1	Thaumatin-like protein	1886.4	MSLLERKLLMHFLRV	4.2–67.9	*Lippia alba*	^58^
La-AMP1a	Thaumatin-like protein	1932.4	GLNKLLRKLLMHPSRVG	8.3–66.2	*Lippia alba*	^58^
La-AMP1b	Thaumatin-like protein	2000.5	GLMKLLRELLHMFSRVG	NR	*Lippia alba*	^58^
Lr-AMP1	Serine/threonine kinase protein	1235.6	MRIGLRFVLM	13.0–103.6	*Lippia rotundifolia*	^58^
Lr-AMP1a	Serine/threonine kinase protein	1990.5	GIGALSAKGMRIGLRFVLM	NR	*Lippia rotundifolia*	^58^
Lr-AMP1b	Serine/threonine kinase protein	1781.3	GMASKAMRIGLRFVLM	NR	*Lippia rotundifolia*	^58^
Lr-AMP1c	Serine/threonine kinase protein	1867.3	HGVSGHGMRIGLRFVLM	34.3–68.6	*Lippia rotundifolia*	^58^
Lr-AMP1d	Serine/threonine kinase protein	1777.2	SVAGRAMRIGLRFVLM	36.0	*Lippia rotundifolia*	^58^
Lr-AMP1e	Serine/threonine kinase protein	1933.4	GIAGLLRSFVRMLAKIMGG	8.3–66.2	*Lippia rotundifolia*	^58^
Lr-AMP1f	Serine/threonine kinase protein	1781.3	GSVLRAIMRMFAKLMG	4.5–35.9	*Lippia rotundifolia*	^58^
Lr-AMP1g	Serine/threonine kinase protein	1867.3	GIHGVVRSFMRMLGHLG	4.3–34.3	*Lippia rotundifolia*	^58^
Lr-AMP1h	Serine/threonine kinase protein	1777.2	GSVIRALMRMFARLVG	4.5–36.0	*Lippia rotundifolia*	^58^
Nicotianin-I (Pep6)	Linear AMP	2422.8	KHYSCTRHGYCLACYKRWF	86.0–141.0	Hybrid of *Nicotiana sanderae* and *Nicotiana langsdorffii*	^59^
Pep1	Linear AMP	1394.7	VCPCACCSTPRRV	NR	Hybrid of *Nicotiana sanderae* and *Nicotiana langsdorffii*	^59^
Pep2	Linear AMP	2646.1	EYCSAKSAKPGVHCRSCALVNMYK	NR	Hybrid of *Nicotiana sanderae* and *Nicotiana langsdorffii*	^59^
Pep3	Linear AMP	2760.1	LHSGEGSTCYFFKTNPCCCGTWNCT	NR	Hybrid of *Nicotiana sanderae* and *Nicotiana langsdorffii*	^59^
Pep4	Linear AMP	2316.8	WKCGSTPAPRKYCTCVAKMW	NR	Hybrid of *Nicotiana sanderae* and *Nicotiana langsdorffii*	^59^
Pep5	Linear AMP	2793.3	HRFSYMCFVAQVLNKDYCSCCKF	NR	Hybrid of *Nicotiana sanderae* and *Nicotiana langsdorffii*	^59^
CaThi-F1^c^	Thionin-like AMP	∼7000	AEGCYKELTKPVKCSSSPRCLYDCIAKTKYDGH	∼14	*Capsicum annuum*	^60^
CaThi-F3^c^	Thionin-like AMP	∼7000	KEICCKVPTTPFLCTNCPQCKTLCSKVNYEDGHCFDILSK	∼14	*Capsicum annuum*	^60^
CaDef2.1	Plant defensin	∼5200	ICEALSGNFKGLCLSSRREGFTDGSCIGFRLQCFCTKPCA	16–18	*Capsicum annuum*	^61^
CaDef2.2	Plant defensin	∼6000	SKYFTGLCWTDSSCRKVCIEKDFQDGHCSKIQR	16–18	*Capsicum annuum*	^61^
*Cc*-F2 (*Cc*-Hev)^d^	Chitin-binding peptide	∼6500	LCCSQYGYCGSTRAYCGVGCQSNCGR	NR	*Capsicum chinense*	^62^
CaCDef-like	Defensin-like	∼3300	VPTTPFLCTNDPQCKVNYEDGHCFDILSK	>15	*Capsicum annuum*	^63^
CaCPin-II	Protease inhibitor	∼3700	RLCTNCCAGRKGCNYYSADGTFICEGESDPNNPKA	>13	*Capsicum annuum*	^63^
Pa-AFP1^c^	Plant defensin	∼11570	PGAGSQEERMQGQMEGQDFSHEERFLSMVRE	2–17	*Passiflora alata*	^54^
CaCLTP2	Lipid-transfer protein	∼3500	GQQSCLCGYMKQYVNSPNARKVVGQCGVSVPNC	>14	*Capsicum annuum*	^63^
γ-thionin	Plant defensin	5769.7	QNNICKTTSKHFKGLCFADSKCRKVCIQEDKFEDGHCSKLQRKCLC​TKNC	NR	*Capsicum chinense*	^65^
Cn-AMP1	Linear AMP with extended-structure scaffold	∼880	SVAGRAQGM	9–144	*Cocos nucifera*	^66^
ZaSNK1^e^	Snakin	∼18500	NEEELYAQVSHPPTPSPAPAAAPVHHIVPVDEKECPGLCEVRCSKHSR​PNHCHRVCQTCCRRCRCVPPGTAGNREMCGACYTDMTTHRNAT​K​CP​K​NV​KC​K​V​E​LGIINDKAAWLTVLYQCDGLHKKSKDKYVTGNC​S​G​SCDFYVFAFHSLTHNNHAYGL​D​A​L​I​​​​PL​LPVLDNQR	NR	*Zantedeschia aethiopica*	^67^
ZaSNK2^e^	Snakin	∼14000	AMAGSGIYTITLRLPSQRYCCTPTTTVVSPPRGGRGVPRLRVSAAA​L​T​E​Q​M​T​SFGAEFCDTKCKARCSKASVHDRCLFNCGVCCKECNCVPSG​T​Y​GNKDECPCYRDKVTKDEKK​K​P​KCP	NR	*Zantedeschia aethiopica*	^67^
ZaSNK3^e^	Snakin	∼11000	AGQSPAPAAQPTVPGPAAQPKLPMYGFTEGSLQPQECSGRCTGR​CS​A​T​Q​YKKPCLFFCQKCCAKCLCVPPGTYGNKQFCPCYNDWKTKR​G​GPKCP	NR	*Zantedeschia aethiopica*	^67^
ZaLTP1^e^	Lipid-transfer protein-like	∼12000	ADDPPCSYVKSNLRPCGPYVFGRVAGPSPQCCAAVRQLNRIARTTA​QRRNVCRCLRGLEVEKAAAGRPFSLNAVRSLPARCGVNLGFPITR​​​Y​​VDCNRVP	NR	*Zantedeschia aethiopica*	^67^
ZaLTP2^e^	Lipid-transfer protein-like	∼13000	AIMCGQVYRTLTPCMGYLRSGGKPSPPCCSGVRELVRLAGTTADRQA​T​CRCLKAAAQGLSGNFGPAASLPSSCGVTISYKISTDTDCNKYLAPV​​P​​IP​W​TVIRSRDWTTYVT	NR	*Zantedeschia aethiopica*	^67^
ZaLTP3^e^	Lipid-transfer protein-like	∼12000	TYCPPPTKKPPPKKPPPVKPPPAYPSCPRDTVKLGGCANVLNLLK​VK​LGAPPKETCCPLLKGLTDVEA​A​M​CLCTVLKASIMGINLNLPVDLSL​L​LS​YCGKSVPSGFKCA	NR	*Zantedeschia aethiopica*	^67^
ZaLTP4^e^	Lipid-transfer protein-like	∼21000	QTGCTAMMVDLNPCSNYITGNISAPPVTCCTQLDNLVQTQMGCL​C​A​F​LANGAPFGIPLNLAQVLSLPGACNIKAGPLSQCYGVATTPAGPSP​​V​​AP​​G​PASPAMPSTPGSALAPSGTPATTPMTPSMPNRPSGAGSKTVPGG​​P​​V​​V​​G​S​STRLGTHPLLFLLLVAITSYASSGVGF	NR	*Zantedeschia aethiopica*	^67^
ZaLTP5^e^	Lipid-transfer protein-like	∼18000	SPIITKVTCLPLSRGNNPKSKGGIPALPPVVKPLPVPVVPPIITIPPVV​G​T​V​TCPVDAVKIGSCVDQLGGLVDVQLGEPAANVCCPVLEGLLEAEV​​A​​VC​L​C​TTLQLKLCNLGIYVPVCLKLLSGLKYGGNHDIRQASDALY	NR	*Zantedeschia aethiopica*	^67^
ZaLTP6^e^	Lipid-transfer protein-like	∼14000	YLDCEDDDIYGLQGHCGKAVWVGAARAAPSTDCCHFVKERTSLAC​​V​C​ENVVTPRHEKYISMKKLAYVAGYCGAPLKPGTKLPGPAKALRL​IL​​L​P​RELKQPQGGAWKCSWGFGKCQGKIL	NR	*Zantedeschia aethiopica*	^67^
ZaHev1^e^	Hevein-like	∼16500	EQCGSQAGGALCPGGLCCSRFGWCGDTAAYCDPAQGCQSQCRGGPT​P​T​P​SPPTGGGGSGVGSIVSQSLFDRMLMHRNDAACPARNFYTYNAFI​​SA​​A​N​SFGGFGTTDSEKARSFCGSGVSDVCSSDGSRGEWERE	NR	*Zantedeschia aethiopica*	^67^
ZaLTP7^e^	Lipid-transfer protein-like	∼17000	ASWAPTAESVTCNPYELLPCAGAISGGSPSRECCARLRAQQPCLC​G​Y​A​R​NPNLVRLVNIPKALQVASACKFLQRVFSLDTVVKPLPPVMAYN​​VS​​R​N​LSFFTRIFTQFFDPEGIANAQKSLGLGQEQKDRRFAVILKLWLKRI​K​H​W​HVLSLAA	NR	*Zantedeschia aethiopica*	^68^
ZaβB1^e^	β-barrelin	∼9300	EGSYMTAWDGPGCNNSAERYSACGCSSINLHGGYEFVYQGQTAAT​YNQPDCQGNNFSGSKLVNIPATRQIVRIISRGNL	NR	*Zantedeschia aethiopica*	^68^
CF15	Snakin	7006.4	LRPRECGGRCTARCSATQYKKPCLEFCNKCCAKCLCVPPGTYGNKQ​V​C​P​CYNWKTKRGGPKCP	NR	*Cereus fernambucensis*	^149^
CH207	Snakin	6865.1	RPQECGGRCTARCSATQYKKPCLEFCNKCCAKCLCVPPGTYGNKQ​​V​C​PCYNWKTKRGGPKCP	NR	*Cereus hildmannianus*	^149^
CJ217	Snakin	6978.3	LRPQECGGRCTARCSATQYKKPCLEFCNKCCAKCLCVPPGTYGNKQ​​V​C​P​CYNWKTKRGGPKCP	NR	*Cereus jamacaru*	^149^
CH167	Snakin	6812.1	LLGINCGAACVARCRLSKRPNLCKRACGTCCARCGCVPPGTSGNQE​L​C​P​CYYSRTTRGGRRKCP	NR	*Cereus hildmannianus*	^149^
CF267	Snakin	6885.0	PSGWCGQKCGVRCSKAGKQARCLRYCLMCCDCRCVPSGTYGNKDE​​C​P​C​RYDWTSPNGRPKCP	NR	*Cereus fernambucensis* and *C. jamacaru*	^149^
CJ149	Snakin	5545.5	PSGWCGQKCGVRCSKAGKQARCLRYCLMCCDCRCVPSGTYGNKD​EC​P​​CRY	NR	*Cereus jamacaru*	^149^

a Molecular mass in Da correspond to the mature bioactive peptide, reported or calculated for the modified form when explicitly stated.

b MIC ranges in μM summarize antimicrobial activity reported against bacteria and/or fungi using broth microdilution or equivalent assays.

c The reported sequence corresponds to the *N*-terminal region, and the molecular mass does not correspond to this sequence but rather to that of the peptide-enriched fraction visualized by gel electrophoresis.

d The reported sequence corresponds to part of a cysteine-rich signature region characterized by a repeating pattern of cysteine residues, whereas the molecular mass refers to the peptide-enriched fraction visualized by gel electrophoresis.

e Predicted AMP Sequences NR, not reported.

Among early diverging eudicots, the defensin identified Pa-AFP1 from *Passiflora alata*,^54^ studied in Brazil, exhibits antifungal activity against the phytopathogen *Colletotrichum gloeosporioides*. These findings reinforce the evolutionary conservation of cysteine-rich peptide scaffolds as key plant defense molecules. This observation supports the notion that disulfide-stabilized peptides represent ancient and functionally robust antimicrobial frameworks. Notably, this structural bias toward disulfide-rich peptides may reflect both evolutionary constraints and methodological preferences, potentially limiting the exploration of alternative scaffold classes with distinct pharmacodynamic profiles.

Within the rosid clade, cysteine-rich defensins from *Phaseolus vulgaris* (PvD1 and recombinant PvD1r) have been investigated and exhibit enhanced potency, particularly against yeast strains,^55,56^ demonstrating the utility of recombinant expression platforms for the production of AMPs. Hevein-like and chitin-binding peptides further illustrate this mechanistic diversity. Similarly, the glycine-rich peptide Pg-AMP1 ^57^ from *Psidium guajava* was structurally and functionally characterized, where the investigations demonstrated moderate micromolar activity, consistent with flexible scaffolds that likely promote membrane destabilization through amphipathic interactions.Despite their structural diversity, the relatively modest potency of several rosid-derived peptides raises important questions regarding their direct therapeutic applicability without further structural optimization or formulation strategies.

Moving toward more derived asterid lineages, exploration of leaf transcriptomes from native and cultivated flora has identified two AMPs, La-AMP1 and Lr-AMP1, in *Lippia alba* and *Lippia rotundifolia*, respectively. Using an *in silico* screening approach followed by synthesis and experimental validation, eight candidate peptides were evaluated against Gram-positive and Gram-negative bacteria; only La-AMP1 and Lr-AMP1 exhibited significant antimicrobial activity. La-AMP1 showed higher potency against *Staphylococcus aureus* MRSA, with a MIC of 4.2 μM, and additional activity against *Pseudomonas aeruginosa* (17.0 μM), while Lr-AMP1 was more active against *Escherichia coli* (MIC of 13.0 μM). Despite their broad-spectrum antibacterial activity, both peptides exhibited high hemolytic activity (52% and 71.5%, respectively), which limits their therapeutic applicability.^58^

Also, within asterid lineages, recently in Brazil, six AMPs were identified through a proteomic analysis that mapped nearly 800 sequences in the floral nectar of ornamental tobacco, a hybrid of *Nicotiana sanderae* and *Nicotiana langsdorffii*. Among these AMPs, nicotianin-I (KHYS­CTRH­GYCL­ACYK­RWF) stood out as the only peptide capable of inhibiting all clinically relevant yeast species tested. Specifically, this peptide exhibited IC_50_ and MIC values as follows 80 μM and 120 μM for *Candida albicans*, 57 μM and 133 μM for *Candida krusei*, 46 μM and 141 μM for *Candida parapsilosis*, and 24 μM and 86 μM for *Candida tropicalis*. The mechanism of action identified for this peptide involved disruption of cytoplasmic membrane integrity and the induction of oxidative stress through the production of reactive oxygen species (ROS) in fungal cells. These observations indicate that nicotianin-I operates through complementary mechanisms involving membrane disruption and oxidative stress induction. The other five peptides (Pep1 to Pep5) did not show significant activity against these clinically relevant yeast species.^59^

A broader repertoire of cysteine-rich peptides, such as defensins and defensin-like, lipid transfer proteins, protease inhibitors and thionin-like peptides from *Capsicum annuum* (CaThi-F1, CaThi-F3, CaDef2.1, CaDef2.2, CaCDef-like, CaCPin-II, CaCLTP2) and *Capsicum chinense* (*Cc*-Hev, CcDef3), were reported to display activity against yeast strains,^60–64^ These peptide studies expand the diversity of disulfide-stabilized AMPs and suggest multiple mechanisms of action, including membrane disruption and intracellular targeting. In addition to their antimicrobial properties, some plant-derived AMPs have been reported to exhibit cytotoxic activity against cancer cell lines, reflecting their broader bioactivity spectrum. For instance, γ-thionin (QNNI­CKTT­SKHF­KGLC­FADS­KCRK­VCIQ­EDKF­EDGH­CSKL­QRKC­LCTK­NC), a cysteine-rich peptide isolated from *Capsicum chinense*, has demonstrated selective cytotoxicity against human cancer cell lines without affecting peripheral blood mononuclear cells (PBMCs). Mechanistically, this peptide induces caspase-independent apoptosis mediated by calpain activation and mitochondrial dysfunction, along with epigenetic modulation through increased histone H3 acetylation and methylation. Although primarily investigated in an oncological context, these findings reveal alternative mechanisms of action, including membrane-independent pathways and intracellular targeting, that may inspire the design of anti-infective peptides with novel modes of action. Unlike the membrane-targeting mechanisms that predominate among many Latin American AMPs, γ-thionin represents a distinct intracellular-targeting peptide whose activity is associated with mitochondrial dysfunction, calcium mobilization, calpain-mediated apoptosis, and epigenetic modulation rather than direct membrane disruption.^65^ Nevertheless, the mechanistic characterization of these peptides remains largely inferred rather than experimentally validated in microbial systems, representing a critical gap that limits their pharmacological positioning.

In monocotyledonous lineages, Cn-AMP1 (∼880 Da), an extended-structure scaffold peptide from *Cocos nucifera*, represents a low-molecular-weight AMP with moderate antimicrobial activity (MICs 9–144 μM).^66^ While these findings illustrate the chemical diversity of plant-derived AMPs, the relatively modest potency observed raises critical questions regarding their direct therapeutic applicability without structural optimization or formulation strategies. Complementarily, research conducted in Brazil has also extended to ornamental plant species, demonstrating the utility of transcriptomic approaches for AMP discovery. In *Zantedeschia aethiopica* spathe transcriptomes studies reported the identification of 12 predicted AMPs, (seven LTPs (ZaLTP1–7), three snakins (ZaSnakin1–3), one hevein (ZaHev1), and one β-barrelin),^67,68^ two of these being never described before. Despite the lack of *in vitro* experimental validation, these findings represent a promising source of new structural scaffolds. These results emphasize that Latin American flora constitute a rich, yet underexploited, reservoir of antimicrobial peptides. Future efforts should prioritize integrative pipelines combining omics-driven discovery, high-throughput functional screening, mechanistic validation, and structure-based optimization to bridge the gap between molecular discovery and clinical applicability. Moreover, fostering regional collaborations and standardizing experimental frameworks will be essential to enhance reproducibility, comparability, and ultimately, the translational impact of plant-derived AMPs from this biodiversity-rich region.

### 2.3 Animal-Derived AMPs Terrestrial and Marine Sources

In addition to plants, animals represent another major source of structurally and functionally diverse AMPs. They have been identified across a wide range of taxa, highlighting their structural diversity and functional relevance as host defense molecules. In Latin America and the Caribbean, both terrestrial and marine organisms represent important sources of these bioactive compounds. Marine invertebrates, including cnidarians, mollusks, and crustaceans, have yielded a variety of AMPs with antimicrobial activity. Arthropods, encompassing arachnids and insects, are also well-established sources of antimicrobial peptides. In addition, vertebrates, such as fish and amphibians, further expand the repertoire of AMPs described in the region. Together, these organisms illustrate the breadth of natural sources explored for the discovery of novel AMPs (Table 3).

**Table 3:** Antimicrobial Peptides from Animal Species in Latin America and the Caribbean Described or Characterized in Primary Studies, Encompassing Marine and Terrestrial Organisms such as Cnidarians, Mollusks, Crustaceans, Arthropods, Fish, and Amphibians

Peptide name	Family/Class	^a^Molecular mass	Sequence	^b^MIC range	Specie	Ref.
Pd-AMP1^c^	NR	5372.7	AKIPGIDQPGCNRQCDVDNDNCCSGYTCQ	NR	*Phyllogorgia dilatata*	^69^
AMP-Ad2	β-sheet-forming peptide	5258.0	MEAFLKLEKIGEGTYGVVYKAKDKENGRTVALKKIRLDTESDGVP​STA	NR	*Acropora digitifera*	^70^
AMP-Ad3	α-helical and β-sheet peptides	4265.9	NGTLVLVGAVSWGFGCAKEGKPGVYTNLIPLRSWIDDIMS	NR	*Acropora digitifera*	^70^
AMP-Ad6	α-helical peptides	4837.6	YINVARFQDRAKVQQICSSLGANLPIIRNSGERDFIFKLLKN	NR	*Acropora digitifera*	^70^
AMP-Ad12	α-helical peptides	6176.9	KYSTCEKAVKSWYSEEKDYNYDHPASSTGVIGHFTQVVWKGSKQL​G​V​G​LVAKRDP	NR	*Acropora digitifera*	^70^
AMP-Ad15	β-sheet-forming peptide	4320.9	CPDGSMCPDSSTCCGVSGGGYGCCPLPDAVCCSDLIHCCPNGY	NR	*Acropora digitifera*	^70^
Pom-1	α-helical peptides	3463.2	KCAGSIAWAIGSGLFGGAKLIKIKKYIAELGGLQ	NR	*Pomacea poeyana*	^71^
Pom-1	α-helical peptides	3603.2	KEIERAGQRIRDAIISAAPAVETLAQAQKIIKGG	NR	*Pomacea poeyana*	^71^
Nv-p1	Linear AMP	1864.1	SGRGKGGKGLGKGGAKRHR	54	*Nerita versicolor*	^73^
Nv-p2	Linear AMP	1707.9	SGRGKGGKGLGKGGAKRH	59	*Nerita versicolor*	^73^
Nv-p3	Linear AMP	857.1	KKKPTKK	116	*Nerita versicolor*	^73^
Litvan ALF-E33-52	α-helical peptides from β-hairpin of ALF	2512.9	YVNRSPYLKKFEVHYRADVK	2.5–40	*Litopenaeus vannamei*	^74^
Litvan ALF-F31-50	α-helical peptides from β-hairpin of ALF	2442.9	TYFVTPKVKSFELYFKGRMT	2.5–40	*Litopenaeus vannamei*	^74^
Litvan ALF-G35-54	α-helical peptides from β-hairpin of ALF	2649.1	SYSTRPYFLRWRLKFKSKVW	2.5–40	*Litopenaeus vannamei*	^74^
Panusin	β-defensin-like peptide	4259.8	SYVGDCGSNGGSCVSSYCPYGNRLNYFCPLGRTCCRRSYG	∼3	*Panulirus argus*	^75^
Gomesin	Defensin-like peptide	2293.4	Pyr-CRRLCYKQRCVTYCRGR-NH_2_	0.2–12	*Acanthoscurria gomesiana*	^76^
LyeTx I	Linear cytolytic AMP	2830.7	IWLTALKFLGKNLGKHLAKQQLAKL-NH_2_	3.8–26.3	*Lycosa erythrognatha*	^77^
Stigmurin	Linear AMP (scorpion AMP)	1796.2	FFSLIPSLVGGLISAFK	8.7–69.5	*Tityus stigmurus*	^81^
Hadrurin	Linear AMP (scorpion AMP)	4436.0	GILDTIKSIASKVWNSKTVQDLKRKGINWVANKLGVSPQAA	8.0–40.0	*Hadrurus aztecus*	^82^
HgeScplp1	Peptide with disulfide bonds – Scorpine-like peptides	∼10400	MNTKLTVLCFLGIVTIVSCGWMSEKKVQGILDKKLPEGIIRNAAKAIV​H​K​M​AKNQFGCFANVDVKGDCKRHCKAEDKEGICHGTKCKCGV​P​ISYL	∼0.5	*Hadrurus gertschi*	^84,85^
Hge36	Peptide with disulfide bonds – Scorpine-like peptides	5298.2	VHKMAKNQFGCFANVDVKGDCKRHCKAEDKEGICHGTKCKCGVP​ISYL	NR	*Hadrurus gertschi*	^84,85^
HgeD	Peptide with disulfide bonds – Scorpine-like peptides	4802.6	AKNQFGCFANVDVKGDCKRHCKAEDKEGICHGTKCKCGVPISYL	NR	*Hadrurus gertschi*	^84,85^
Polybia-CP	Mastoparan-like AMP	1230.7	ILGTILGLLKSL-NH_2_	12.2–203	*Polybia paulista*	^89^
Polybia-MP1	Mastoparan-like AMP	1654.1	IDWKKLLDAAKQIL-NH_2_	2.4–9	*Polybia paulista*	^88,89^
Peptide 5065	Linear AMP	977.2	FGVKWVKN	6.25–25	*Seriolella violacea*	^91^
Peptide 5069	Linear AMP	1186.4	SRSALWLRTP	12.5–25	*Seriola lalandi*	^91^
Peptide 5070	Linear AMP	1092.3	LGKFKGRSPC	25	*Seriola lalandi*	^91^
Peptide 5076	Linear AMP	974.2	LGLFKGRSP	100	*Seriola lalandi*	^91^
Dermaseptin B	Dermaseptin (α-helical AMP)	2645.5	DVLKKIGTVALHAGKAALGAVADTISQ-NH_2_	40–65	*Phyllomedusa bicolor*	^92^
Dermaseptin DS-01	Dermaseptin (α-helical AMP)	2793.4	GLWSTIKQKGKEAAIAAAKAAGQAALGAL-NH_2_	3.2–25.7	*Phyllomedusa oreades*	^93^
Dermadistinctins DD-K:	Dermadistinctins (α-helical AMP)	2926.5	ALWKTLLKNVGKAAGKAALNAVTDMVNQ	10–20	*Phyllomedusa distincta*	^94^
Dermadistinctins DD-L:	Dermadistinctins (α-helical AMP)	3152.7	GLWSKIKAAGKEAAKAAAKAAGKAALNAVSEAV	10–20	*Phyllomedusa distincta*	^94^
DShypo-01	Dermaseptin-like	2409.4	GLWSTIKNVGKEAAIAAGKAALGAL-NH_2_	6.6–26.5	*Phyllomedusa hypochondrialis*	^95^
Hylin a1	α-Helical cytolytic AMP	1864.4	IFGAILPLALGALKNLIK	8–67	*Hypsiboas albopunctatus*	^96^
Hs-1	Linear AMP	2144.6	FLPLILPSIVTALSSFLKQG	11–46	*Hypsiboas semilineatus*	^97^
Figainin-1	α-helical AMP	1915.2	FIGTLIPLALGALTKLFK	1–16	*Boana raniceps*	^98^
Figainin-2	α-helical AMP	3005.7	FLGAILKIGHALAKTVLPMVTNAFKPKQ	NR	*Boana raniceps*	^99^

a Molecular masses in Da correspond to the mature bioactive peptide, reported or calculated for the modified form, when explicitly stated.

b MIC ranges in μM summarize antimicrobial activity reported against bacteria and/or fungi using broth microdilution or equivalent assays.

c The reported sequence corresponds to the *N*-terminal region, and the molecular mass does not correspond to this sequence but rather to that of the peptide-enriched fraction visualized by gel electrophoresis. NR, not reported. Pyr is pyroglutamate acid.

Among animal sources, marine invertebrates stand out as an important reservoir of AMPs. One of the earliest examples reported in Brazil is Pd-AMP1, identified from protein extracts of the gorgonian coral *Phyllogorgia dilatata*. These extracts inhibited more than 50% of the tested bacterial species at a concentration of 100 μg·mL^–1^. Pd-AMP1 was subsequently isolated through chromatographic purification and characterized by mass spectrometry, with its *N*-terminal sequence determined by Edman degradation.^69^

In Colombia, *in silico* identification of AMPs was performed by using transcriptomic data from the coral *Acropora digitifera*. Computational approaches, including multiple sequence alignment, hidden Markov models, and machine-learning algorithms, were applied to predict antimicrobial activity. The sequences were obtained from different life stages of the coral and *in vitro* cultured cells. A total of 15 sequences with potential antimicrobial activity were identified, of which five (AMP-Ad2, AMP-Ad3, AMP-Ad6, AMP-Ad12, and AMP-Ad15) were selected for further analysis due to their homology to scolopendin, lactose-binding lectin I-2, TCP, and SHK toxin, respectively. The activity of these AMPs was evaluated *in silico* against molecular targets from Gram-negative microorganisms, including *Klebsiella pneumoniae*, *Pseudomonas aeruginosa*, and *Escherichia coli*, with a focus on their interaction with bacterial resistance-related targets, specifically the efflux channels TolC and OprM, as well as the enzyme DNA gyrase B. Molecular docking analyses revealed that AMP-Ad2 showed the most favorable interaction with the TolC channel of *E. coli*, while AMP-Ad3 exhibited a higher affinity for OprM from *P. aeruginosa*. AMP-Ad15 demonstrated the strongest interaction observed in the study with DNA gyrase B from *K. pneumoniae*.^70^

Mollusks have emerged as a relevant source of AMPs across both freshwater and marine environments. For instance, a study conducted by Cuban research groups in collaboration with German institutions identified two AMPs, Pom-1 (KCAG­SIAW­AIGS­GLFG­GAKL­IKIKK­YIAE­LGGLQ) and Pom-2 (KEIE­RAGQ­RIRD­AIISA­APAV­ETLA­QAQK­IIKGG), from the freshwater snail *Pomacea poeyana* using LC-MS/MS combined with bioinformatics prediction tools, including CAMPR3, AMP Scanner v2, and iAMPpred. Pom-1 exhibited strong antibacterial activity in antibiogram-type assays, particularly against *Pseudomonas aeruginosa*, and moderate activity against *Klebsiella pneumoniae* and *L. monocytogenes*, whereas Pom-2 showed lower activity. Both peptides also displayed low cytotoxicity toward human macrophages.^71^

Extending this diversity to marine systems, several studies have reported AMPs from Caribbean mollusks collected along the Cuban coast. Protein and peptide fractions from the gastropod *Cenchritis muricatus* showed antibacterial effects, with high-molecular-weight fractions (>10 kDa) inhibiting microbial growth at 100 μg·mL^–1^, reducing *Staphylococcus aureus* growth by 8–10% and *Escherichia coli* by 12–24%.^72^ In parallel, three AMPs, Nv-p1 (SGRG­KGGK­GLGK­GGAK­RHR), Nv-p2 (SGRG­KGGK­GLGK­GGA­KRH), and Nv-p3 (KKKPTKK), were identified in the marine snail *Nerita versicolor*, collected at Jibacoa Beach in Havana (Cuba). All peptides exhibited MIC values above 100 μg·mL^–1^, corresponding to 54 μM (Nv-p1), 59 μM (Nv-p2), and 116 μM (Nv-p3), against *Pseudomonas aeruginosa*, *Candida albicans*, *Candida parapsilosis*, and *Candida auris*. Nv-p2 and Nv-p3 reduced biofilm formation by approximately 50% in the tested *Candida* species, whereas Nv-p1 showed a similar effect only against *C. parapsilosis*, with no significant activity against *C. albicans* biofilms.^73^

Beyond mollusks, arthropods represent another major lineage of AMP-producing organisms. For exemple, a collaborative study conducted by researchers from Brazil and Chile, three linear peptides, litvan ALF-E33–52 (YVNR­SPYL­KKFE­VHYR­ADVK), litvan ALF-F31–50 (TYFV­TPKV­KSFE­LYFK­GRMT), and litvan ALF-G35–54 (SYST­RPYF­LRWR­LKFK­SKVW), were designed based on the amino acid sequence of the central β-hairpin of antilipopolysaccharide factors (ALF) from *Litopenaeus vannamei*. These α-helical peptides exhibit broad-spectrum antibacterial activity against Gram-positive and Gram-negative bacteria, including multidrug-resistant isolates, as well as antifungal activity, synergistic effects, and the ability to permeabilize bacterial membranes. Notably, litvan ALF-G35–54 demonstrated the broadest and most potent antibacterial activity (2.5–40 μM), inhibiting the growth of most tested bacterial strains, including clinical isolates of methicillin-resistant *Staphylococcus aureus* (MRSA) 16003 (cefoxitin-resistant) and 17022 (resistant to cefoxitin, ciprofloxacin, clindamycin, erythromycin, gentamicin, rifampicin, and trimethoprim–sulfamethoxazole). This peptide also exhibited antifungal activity, completely inhibiting spore germination of *Fusarium oxysporum* MUCL 909, *Rhizopus* sp. LAMPB-UFSC, and *Penicillium* sp. LIAA-UFSC (a strain associated with the shrimp midgut). Importantly, these peptides also displayed low cytotoxicity toward human THP-1 cells and retained activity against multidrug-resistant bacterial isolates, supporting the potential of ALF-derived peptides as selective anti-infective scaffolds with favorable therapeutic profiles.^74^

Within arthropods, venom-derived peptides have attracted particular attention due to their potent bioactivities. One example is panusin, a β-defensin-like peptide identified in hemocytes of the spiny lobster *Panulirus argus*, collected from the Batabanó Gulf in the southwestern Cuban archipelago, and investigated by Cuban research groups in collaboration with researchers from Spain and Belgium. Panusin exhibited broad-spectrum antimicrobial activity against *Escherichia coli*, *Klebsiella pneumoniae*, *Staphylococcus aureus*, *Bacillus subtilis* subsp. *spizizenii*, and *Candida albicans*, with vLD90 values below 3 μM.^75^

Arthropods, in turn, have a guaranteed place as excellent representatives of Latin America’s biodiversity and, unsurprisingly, also emerge as a powerful source of AMPs. A prominent representative of this group is gomesin (Pyr-CRRLC­YKQR­CVTYC­RGR-NH_2_),^76^ isolated from hemocytes of the spider *Acanthoscurria gomesiana* from a Brazilian specimen. This 18-residue cysteine-rich peptide (β-hairpin defensin-like peptide stabilized by two disulfide bonds) is structurally related to tachyplesins and displays potent activity (MICs 0.2–12 μM), reflecting enhanced proteolytic stability and defined membrane interactions against bacteria, fungi, and parasites. The disulfide-stabilized β-hairpin scaffold provides conformational rigidity and resistance to enzymatic degradation, structural features that contribute directly to the potent antimicrobial activity of those peptides. Similarly, other studies conducted in Brazil have yielded remarkable results with venom-derived peptides, such as LyeTx I (IWLTA­LKFLG­KNLG­KHLA­KQQL­AKL-NH_2_, MICs 3.8–26.3 μM),^77^ from *Lycosa erythrognatha*, further illustrating the antimicrobial potential of toxin-derived scaffolds. LyeTx I exhibits potent membrane-targeting antimicrobial activity and has inspired the development of optimized analogs with enhanced *in vivo* efficacy. Notably, the shortened derivative LyeTx I mnΔK (IWLTK­ALKF­LGKN­LGK-NH_2_), reduced bacterial burden in a murine septic arthritis model, an effect accompanied by decreased inflammatory cell recruitment and attenuation of infection-associated pain.^78^ These findings suggest that LyeTx-derived peptides may provide therapeutic benefits that extend beyond direct microbial killing and contribute to improved infection outcomes.

Within arachnids, scorpion venoms represent a rich source of bioactive peptides with diverse biological activities, including antimicrobial, antiparasitic, and ion-channel-modulating properties. Beyond linear cytolytic peptides, scorpion venoms also harbor cysteine-rich antimicrobial peptides with distinct structural features, among which defensins and scorpine-like peptides are prominent representatives. These peptides are typically classified into two major groups including short-chain peptides containing approximately 23–50 amino acids and longer toxins containing 53–78 amino acids. Many of these molecules adopt structurally stable conformations through amphipathic α-helices or cysteine-stabilized motifs formed by disulfide bridges, which contribute to their biological activity.^79,80^

Several antimicrobial peptides derived from scorpion venoms have been characterized by research groups in Latin America, including stigmurin (FFSL­IPSLV­GGLI­SAFK, MICs 8.7–69.5 μM),^81^ identified through transcriptomic analysis of the Brazilian scorpion *Tityus stigmurus*. Other linear AMP lacking disulfide bonds, hadrurin (GILD­TIKS­IASK­VWNS­KTVQ­DLKR­KGIN­WVAN­KLGV­SPQAA), a 41-residue peptide isolated from the venom of the scorpion *Hadrurus aztecus*, from Mexico, exhibits antimicrobial activity against a variety of bacterial pathogens, including *Salmonella typhi*, *Klebsiella pneumoniae*, *Pseudomonas aeruginosa*, *Escherichia coli*, and *Serratia marcescens* (MICs 8–40 μM). Although hadrurin exhibits broad antimicrobial activity, it also shows hemolytic activity against human erythrocytes, underscoring the need to balance antimicrobial potency with host cytotoxicity in venom-derived peptides.^82^

Among scorpion-derived peptides, a distinct group known as scorpine-like peptides combines structural and functional features of AMPs and ion-channel-active toxins. These peptides typically contain two functional domains: an *N*-terminal amphipathic α-helix associated with antimicrobial activity and a *C*-terminal cysteine-rich domain that forms a cysteine-stabilized αβ (CSαβ) motif via three disulfide bonds.^83^ Detailed structural studies have been performed on scorpine-like peptides isolated from the Mexican scorpion *Hoffmannihadrurus gertschi* (formerly *Hadrurus gertschi*). One example is the peptide HgeScplp1, which exhibits antibacterial activity against *Bacillus subtilis* (MIC 0.5 μM) but shows no activity against *Staphylococcus aureus*. Interestingly, HgeScplp1 also induces lysis of eukaryotic cells, including oocytes and red blood cells, at all concentrations.^84^ Truncated variants derived from HgeScplp1 have also been characterized. Removal of 27 residues from the *N*-terminus generates Hge36 (VHKM­AKNQ­FGCF­ANVD­VKGD­CKRH­CKAE­DKEG­ICHG­TKCK­CGVP­ISYL), whereas removal of 31 residues produces HgeD (AKNQ­FGCF­ANVD­VKGD­CKRH­CKAE­DKEG­ICHG­TKCK­CGVP­ISYL). These truncated peptides exhibit distinct biological activities, particularly as antiparasitic. Molecular dynamics studies carried out on both peptides by three-dimensional NMR experiments showed that the *C*-terminal fraction of both peptides is structured by the formation of α-helix and two β-sheets stabilized by three disulfide bonds, typical of the motif CSαβ of scorpine-like peptides. Both motifs, once superimposed, remain aligned between amino acids 5–47 in Hge36 and 2–43 in HgeD, and there is a well-marked structural difference in both by the presence and absence of the four residues (VHKM) in the *N*-terminal region. NOE interactions enabled determination of the *N*-terminal region in both peptides. In HgeD, the absence of the lysine residue results in a different charge distribution, positioning the *N*-terminus closer to the CSαβ core and increasing the local positive charge in this region. In contrast, the *N*-terminus of Hge36 is positioned further away from the CSαβ motif. This difference in charge distribution likely explains why HgeD displays two- to 4-fold greater activity than Hge36 against cysticerci and amoebic stages.^85^ These peptides exert their antiparasitic effects mainly through the induction of apoptosis in specific parasite structures, such as the bladder wall and the scolex of the cysticerci. The underlying mechanism may involve the blocking of ion channels, particularly potassium channels, which are known targets of peptides containing CSαβ motifs stabilized by three disulfide bonds.^84^ More broadly, the biological activities of venom-derived peptides are frequently associated with their interactions with ion channels. Ion channels play essential roles in maintaining cellular homeostasis, regulating membrane potential, intracellular pH, and cell survival. In parasites such as *Trypanosoma cruzi*, potassium channels have been identified as potential pharmacological targets, illustrating the relevance of venom-derived peptides as molecular tools for the development of antiparasitic therapies.^86,87^

In a study on social insects, a research group based in Brazil reported the mastoparans polybia-MP1 (IDW­KKLL­DAAK­QIL-NH_2_, MICs 2.4–9.1 μM) and polybia-CP (ILGT­ILGL­LKSL-NH_2_, MICs 12.2–203 μM) from *Polybia paulista*. Polybia-MP1, the more active peptide, exhibits selective membrane disruption, preferentially targeting negatively charged lipid bilayers, supporting its antimicrobial activity against Gram-positive and Gram-negative bacteria.^88,89^ Beyond their direct antimicrobial effects, these venom-derived peptides also display immune-related functions. Polybia-MP1 was characterized as a mast cell lytic peptide with chemotactic activity toward polymorphonuclear leukocytes (PMNL), whereas polybia-CP exhibited chemotactic activity without significant mast cell degranulation at physiological concentrations.^89^ These findings indicate that polybia-derived peptides combine antimicrobial and inflammatory cell-recruitment properties, illustrating how certain venom-derived AMPs may contribute to host defense through mechanisms extending beyond direct pathogen killing.^88,89^

Marine vertebrates have also been identified as relevant sources of AMPs in Latin American waters, particularly teleost fish. Studies on species captured as bycatch along the Brazilian coast, including *Paralonchurus brasiliensis* and *Micropogonias furnieri*, have reported short AMPs (4–12 amino acid residues) derived from muscle protein hydrolysates. Among these, hydrolysates obtained from *P. brasiliensis* muscle using Protamex showed potent antifungal activity, inhibiting the growth of *Candida albicans* by 81%, whereas those produced using alcalase exhibited moderate antibacterial activity against *Staphylococcus aureus* (29% inhibition), both evaluated at 250 μg·mL^–1^.^90^

Building on these findings, more detailed peptidomic analyses of marine fish from the Pacific coast of Chile, combined with bioinformatics tools and antimicrobial assays, have further expanded the repertoire of vertebrate-derived AMPs. In particular, peptides identified in the epidermal mucus of *Seriola lalandi* and *Seriolella violacea* revealed several promising candidates, including peptide 5065 (FGVK­WVKN) from *S. violacea* and peptides 5069 (SRSAL­WLRTP), 5070 (LGKFK­GRSPC), and 5076 (LGLF­KGRSP) from *S. lalandi*. Peptide 5065 exhibited MIC values of 6.25 μM against *Vibrio ordalii*, 12.5 μM against *Vibrio anguillarum*, and 25 μM against *Escherichia coli*. Peptide 5069 showed MIC values of 12.5 μM against both *Vibrio* strains and 25 μM against *E. coli*. Finally, peptides 5070 and 5076 displayed identical MIC values of 25 μM against all tested bacteria, except that peptide 5076 showed no activity against *V. anguillarum* at the highest concentration tested (100 μM).^91^

**A**mphibian skin secretions remain a prominent source of helical peptides, particularly dermaseptins and dermaseptin-like analogs isolated from *Phyllomedusa* species. An early example is dermaseptin B (DVLK­KIGT­VALH­AGKA­ALGA­VAD­TISQ-NH_2_), first described in 1994 from the skin of the Amazonian frog *Phyllomedusa bicolor* from Guiana region. It is a 27-residue amphipathic peptide displaying moderate antimicrobial activity, illustrating the early discovery of dermaseptin-type host-defense peptides in Neotropical amphibians.^92^ Studies from Brazil have demonstrated that dermaseptins (DS) and dermadistinctins (DD) isolated from *Phyllomedusa oreades*, DS-01: GLWS­TIKQ­KGKE­AAIA­AAKA­AGQA­ALGAL-NH_2_, (MICs 3.2–25.7 μM),^93^ *P. distincta* (DD-K: ALWK­TLLK­NVGK­AAGK­AALN­AVTD­MVNQ and DD-L: GLWS­KIKA­AGKE­AAKA­AAKA­AGKA­ALNA­VSEAV, MICs 10–20 μM),^94^ and *P. hypochondrialis*, DShypo-01: GLWS­TIKN­VGKE­AAIA­AGKA­ALGAL-NH_2_ (MICs, 6.6–26.5 μM)^95^ exhibit broad-spectrum membrane-disruptive activity against bacteria and fungi. In addition, DS-01 and dermadistinctins demonstrated potent anti-*Trypanosoma cruzi* activity with limited toxicity toward mammalian blood cells,^93,94^ whereas DShypo-01 showed activity against *Leishmania amazonensis* and favorable selectivity profiles in mammalian-cell assays.^95^ The ability of these peptides to target bacterial, fungal, and protozoan pathogens illustrates the remarkable breadth of anti-infective activities present within amphibian host-defense systems.

Similarly, studies in Brazil showed that hylin-A1 (IFGA­ILPL­ALGA­LKNLIK, MICs 8–67 μM), from *Hypsiboas albopunctatus*,^96^ and the related peptide HS-1 (FLPL­ILPSI­VTAL­SSFL­KQG, MICs 11–46 μM) from *Hypsiboas semilineatus*, which stands out for its activity against Gram-positive strains,^97^ expand the repertoire of frog-derived cytolytic peptides with antibacterial and antifungal properties. More recently, figainins,^98,99^ from *Boana raniceps*, demonstrated antimicrobial and antiproliferative activities, reinforcing amphibian skin as a reservoir of multifunctional bioactive scaffolds. Figainin-1 (FIGTL­IPLA­LGAL­TKLFK, MICs 1–16 μM) and its precursor-derived counterpart, Figainin-2 (FLGAI­LKIG­HALA­KTVL­PMVT­NAFK­PKQ), described as a broad-spectrum host defense peptide active against Gram-negative and Gram-positive bacteria, as well as emerging arboviruses, further illustrate the evolutionary diversification of frog host-defense peptides. Beyond their antibacterial activity, figainin peptides also exhibit additional biological activities. Figainin-1 demonstrated activity against *Trypanosoma cruzi* and antiproliferative effects against multiple tumor cell lines, whereas figainin-2 displayed antiviral activity against emerging arboviruses in addition to its antibacterial properties. These findings further illustrate the broad biological repertoire of amphibian host-defense peptides and their potential for broader anti-infective and biomedical applications. These cationic peptides, rich in hydrophobic residues, adopt an amphipathic helical structure in the presence of the helix-inducer TFE, with figainin-1 maintaining low-micromolar potency.^98,99^

These studies highlight Latin American biodiversity as a prolific source of animal AMPs with distinct structural motifs, including helical, hairpin, and disulfide-stabilized frameworks, which make them capable of targeting multidrug-resistant pathogens. Their mechanistic diversity and tunable selectivity position them as promising templates for the next generation of anti-infective development.

A broader view of the peptides discussed herein reveals a broad mechanistic spectrum extending beyond the traditional paradigm of membrane disruption. Membrane-associated activity remains predominant among numerous Latin American AMPs, including amphibian peptides such as dermaseptins and figainins, microbial lipopeptides such as pelgipeptins, crustacean ALF-derived peptides, marine-derived AMPs, venom-derived peptides such as polybia-MP1 and LyeTx-I, and fungal peptaibols such as trilongins, which primarily exert their antimicrobial effects through membrane permeabilization, ion-channel formation, membrane depolarization, or disruption of membrane integrity.^100,101^ However, several examples demonstrate that AMP activity in organisms from Latin American is not limited to direct membrane targeting. Nicotianin-I combines membrane damage with oxidative stress induction, representing a dual mechanism that contributes to fungal killing through complementary cellular effects, whereas peptide L3 promotes membrane depolarization together with nucleoid relaxation and alterations in DNA organization, suggesting mechanistic effects that extend beyond membrane perturbation. These observations are consistent with growing evidence that AMP activity frequently involves multiple cellular targets and stress-response pathways in addition to membrane interactions. In contrast, γ-thionin from *Capsicum chinense* represents a distinct intracellular-targeting peptide, inducing mitochondrial dysfunction, calcium mobilization, calpain-mediated apoptosis, and epigenetic modulation without detectable disruption of plasma membrane integrity.^102,103^ From a translational perspective, membrane-targeting AMPs generally provide rapid microbicidal activity and potent antibiofilm effects, whereas intracellular-targeting and multifunctional peptides may offer additional opportunities to overcome resistance through alternative cellular pathways and multitarget modes of action.^102,103^ This mechanistic diversity provides multiple opportunities for peptide engineering strategies aimed at expanding antimicrobial efficacy while addressing different biological targets.

Beyond mechanistic diversity, an important trend emerging from studies of Latin American AMPs is that the antimicrobial potency alone may not fully predict their therapeutic relevance. While most peptides were initially characterized for their microbicidal activity, growing evidence suggests that additional biological attributes can contribute to the anti-infective efficacy. These include activity against multiple classes of pathogens, antibiofilm effects, favorable selectivity toward host cells, and, in some cases, modulation of infection-associated inflammatory responses. Rather than representing isolated biological features, these properties may collectively enhance therapeutic outcomes by improving pathogen control while reducing the treatment-associated limitations. Consequently, future AMP development may benefit from integrating these complementary attributes into peptide selection and optimization strategies, particularly in the context of antimicrobial resistance, where successful interventions often require more than direct microbial killing alone.^104–106^

## 3 Rational Engineering Strategies of AMPs in Latin America

In recent years, research groups across Latin America have progressively incorporated rational engineering strategies to strengthen the therapeutic and biotechnological potential of AMPs. This approach responds to the growing global challenge of antimicrobial resistance by enabling the deliberate refinement of peptide sequences to improve activity, selectivity, stability, and toxicity profiles. Because AMPs often act through mechanisms distinct from those of classical antibiotics, including membrane perturbation and multitarget intracellular interactions, the likelihood of cross-resistance remains comparatively low, reinforcing their relevance for anti-infective innovation.^27,107,108^

The rational development of AMPs increasingly relies on detailed structural and conformational knowledge to optimize their biological performance. Parameters such as conformational rigidity, amphipathicity, charge distribution, secondary-structure propensity, and topological organization are critical determinants of peptide-membrane interactions and intracellular targeting, antimicrobial potency, and selectivity.^29–32^ Accordingly, advances in peptide biosynthesis, including nonribosomal peptide synthetase (NRPS) assembly pathways, cyclization strategies, post-translational modifications, and chemical optimization approaches, have emerged as powerful strategies for generating structurally diverse peptide scaffolds with enhanced stability, selectivity, proteolytic resistance, and therapeutic efficacy.^109–111^ In parallel, chemical optimization approaches and advances in solid-phase peptide synthesis (SPPS) have enabled the rational production and engineering of peptide analogues with improved pharmacological properties, facilitating their translation into clinically relevant therapeutics.^103,112^ Particularly in the case of nonribosomal peptides and engineered AMP derivatives, growing knowledge of biosynthetic pathways combined with modern synthetic methodologies has accelerated the development of next-generation peptide-based anti-infective agents.^111,113^

In this context, efforts in the region have focused on sequence and structural optimization to refine physicochemical parameters such as net charge, hydrophobicity, amphipathicity, and propensity for secondary structure. These strategies are guided by SAR principles, which associate specific molecular features with antimicrobial performance and selectivity, and are implemented through approaches such as amino acid substitution, chemical modification, peptide conjugation, and the design of hybrid or chimeric peptides.

Although many foundational advances in AMP engineering originated outside Latin America, regional research has progressively shifted from descriptive bioprospecting to more structured, hypothesis-driven, and application-oriented design frameworks. This transition has been supported by advances in peptide synthesis, molecular characterization, and biological evaluation, enabling more systematic optimization and an improved translational potential.

Overall, the advances summarized in Figure 2 underscore the consolidation of rational design as a central framework guiding AMP research in Latin America and highlight how structural characterization and rational modification strategies, including cyclization, sequence truncation, PEGylation, and conformational stabilization, can support the development of engineered peptides with improved stability, selectivity, pharmacokinetic performance, reduced toxicity, and enhanced translational anti-infective potential. Recent advances in peptide engineering, delivery technologies, and chemical modification approaches have further accelerated the optimization of AMPs for clinical and biotechnological applications.^103,114–116^

**Figure 2:**
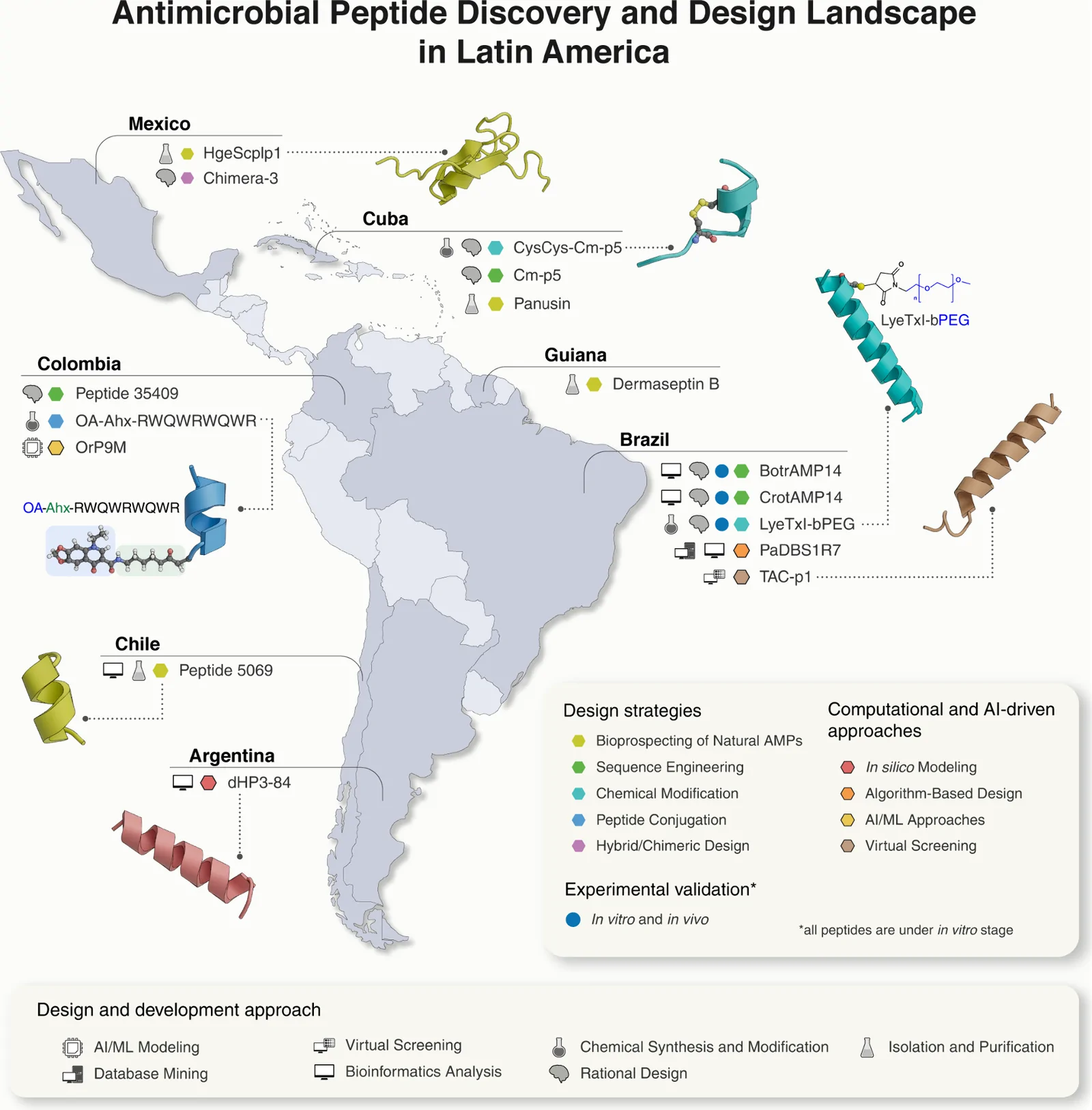
Overview of representative antimicrobial peptides (AMPs) identified or designed in Latin America, categorized according to their geographic origin, design strategies, methodological approaches, and levels of experimental validation. Design strategies encompass the main frameworks for AMP discovery and engineering, including bioprospecting of natural peptides, sequence-based optimization, chemical modification, peptide conjugation, and hybrid or chimeric design. Computational and artificial intelligence (AI) driven approaches are presented as complementary methodologies that support these strategies, including *in silico* modeling, algorithm based design, AI/ML methods, and virtual screening. Experimental validation indicates the stage of biological assessment (*in vitro* and *in vivo*), noting that the peptides represented are currently limited to *in vitro* evaluation. Icons denote representative experimental and computational workflows, such as isolation and purification, chemical synthesis and modification, bioinformatics analysis, database mining, AI/ML modeling, virtual screening, and rational design, which may overlap across categories and are not mutually exclusive.

Research on AMPs in Latin America has historically been grounded in the bioprospecting of native biodiversity and *in vitro* evaluation against clinically relevant pathogens. Although systematic sequence engineering is not yet a predominant regional strategy, there is a growing incorporation of physicochemical criteria, including net positive charge, hydrophobic moments, and amphipathicity, into peptide design frameworks. This trend reflects a gradual transition from primarily descriptive discovery to more structure-informed optimization, supported by the extensive molecular diversity available in the region.

An illustrative example of this emerging approach is a study conducted in Brazil in which rational design strategies were applied to two generations of Hs02 peptide analogues. Hs02 (KWAV­RIIRK­FIKG­FIS-NH_2_), an intragenic AMP identified in human proteins, exhibits broad-spectrum antimicrobial and anti-inflammatory activities.^117^ In the first generation, modifications included terminal truncations (three residues) and segment deletions (six-residue segments), whereas the second generation involved targeted residue substitutions, replacing Phe-14 and Ile-15 residues with alanine to reduce hydrophobicity. These changes aimed to enhance selectivity against Gram-negative bacteria while reducing cytotoxicity toward eukaryotic cells (murine BV-2 microglia). The results demonstrated that subtle structural modifications can significantly improve activity and selectivity, highlighting the potential of sequence-level optimization applied to biological templates.^118^

A similar rationale was applied to peptides from *Lippia alba* and *Lippia rotundifolia.* The initial peptides, La-AMP1 (MSLL­ERKL­LMHF­LRV, MICs 4.2–17.0 μM) and Lr-AMP1 (MRIGL­RFVLM, MIC ∼ 13.0 μM), exhibited antimicrobial activity but were limited by pronounced hemolytic effects. Subsequent modifications generated variants with improved biological profiles, including Lr-AMP 1f (GSVL­RAIM­RMFA­KLMG), Lr-AMP1h (GSVI­RALM­RMFA­RLVG), and Lr-AMP1g (GIHG­VVRS­FMRM­LGHLG), which retained or enhanced antimicrobial activity while markedly reducing hemolytic effects. In particular, Lr-AMP 1f and Lr-AMP1h displayed potent activity against *Staphylococcus aureus* MRSA, with MIC values of approximately 4.5 μM; notably, Lr-AMP 1f showed no detectable hemolysis, whereas Lr-AMP1h exhibited only ∼ 2.3%. Additionally, Lr-AMP1h demonstrated an expanded antimicrobial spectrum, remaining active against *Staphylococcus epidermidis* and *Pseudomonas aeruginosa* at similar concentrations, while Lr-AMP1g also showed relevant activity without detectable cytotoxicity. In contrast, the La-AMP1a variant (GIGALSAKGMRIGLRFVLM) exhibited a substantial reduction in hemolytic activity (from 52% to 0.5%), albeit with some loss of potency. Importantly, although the study does not provide a fully systematic SAR analysis, the results indicate that subtle sequence modifications significantly modulate membrane interactions, thereby improving selectivity and reducing hemolytic activity. Such findings reinforce how rational engineering, guided by structural and physicochemical principles, can effectively refine natural peptide scaffolds, improving the balance between antimicrobial efficacy and host cell compatibility.^58^

Along similar lines, a practical application of rational design and bioinformatics-driven strategies developed by our group is illustrated by the development of BotrAMP14 (KRWK­KFFR­KVIKFF-NH_2_,) and CrotAMP14 (KRLK­KIFKK­MIKIF-NH_2_), both exhibiting MIC values between 1.5 and 12.5 μM. These peptides were redesigned from the cathelicidins of the South American pit vipers, *Bothrops atrox* and *Crotalus durissus terrificus*, through the elimination of unfavorable residues such as Val, Pro, Gly, and Thr to optimize the electrostatic surface. Guided by bioinformatics to identify in optimal motifs; this approach yielded smaller variants with enhanced bactericidal activity and reduced toxicity toward human cells. These peptides adopted helical conformations that favor membrane interaction, illustrating how targeted sequence alterations and computational tools can significantly improve both selectivity and potency.^119^

In Colombia, research groups and various institutions have collaborated to evaluate the *in vitro* activity of synthetic AMPs against standard ATCC strains and clinical isolates of *Candida* spp. resistant to fluconazole and amphotericin, as well as their efficacy against biofilms. The peptide PNR20 was designed by randomly inserting 10 polar and 10 nonpolar residues, yielding its derivative PNR20-1, which features a Lys10-to-Ala substitution. Although the exact sequences of the first two peptides were not disclosed, a third analog, 35409 (RYRR­KKKM­KKAL­QYIK­LLKE; MICs 22–350 μM against bacteria), derived from the *Plasmodium falciparum PfRif* 20628 protein (^321^RYRR­KKKM­KKKL­QYIK­LLKE^340^) via a Lys332-to-Ala substitution, was also evaluated.^120^ All three peptides demonstrated significant antifungal potential, with MIC values ranging from 25 μM to 100 μM. This study reinforces the importance of structural parameters, such as net positive charge and hydrophobicity, in biological activity, illustrating the application of classical AMP principles, specifically amphipathicity and direct interaction with microbial membranes.^121^

Chemical modifications aimed at improving the antimicrobial peptide performance have also been explored by research groups in Latin America. Strategies such as cyclization, PEGylation, incorporation of *D*-amino acids, *N*-methylation, lipidation, and glycosylation have been increasingly applied to optimize the AMP stability and biological activity. These structural modifications can significantly enhance peptide performance by improving solubility and stability in aqueous media, reducing susceptibility to proteolytic degradation and renal clearance, and extending the metabolic half-life. As a result, these approaches can strengthen the antimicrobial activity and broaden the therapeutic potential of AMPs through diverse molecular mechanisms.^26^ Notably, lipidation has been widely reported to enhance membrane affinity and antimicrobial potency, while glycosylation can improve peptide stability, solubility, and biological recognition.^122^

Among these approaches, cyclization has emerged as a particularly effective strategy to enhance structural stability. Representative examples include disulfide-bridged derivates of *Cm*-p5 (SRSE­LIVH­QRLF-NH_2_), formed by cysteine or homocysteine (Hcy), such as cyclics CysCys-*Cm*-p5 (*i+4*) (SRSC­LIVC­QRLF-NH_2_, Figure 1C), HcyHcy-*Cm*-p5 (*i+4*) (SRS-Hcy-LIV-Hcy-QRLF-NH_2_), and HcyCys-*Cm*-p5 (*i+4*) (SRS-Hcy-LIVC­QRLF-NH_2_). In addition, dimeric variants containing two disulfide bonds formed by cysteine residues were also developed, including dimer 1 (parallel) and dimer 2 (antiparallel). The development of these derivatives was motivated by the limited clinical potential of *Cm*-p5, mainly due to its proteolytic instability and relatively lower antifungal activity compared with conventional antifungal drugs.^123,124^ Previously, *Cm*-p5 had been shown to exhibit antifungal activity against the pathogenic yeasts *Candida albicans* (strains 38U and 01U; MIC = 6.7 μM) and *Candida parapsilosis* (MIC = 21.6 μM), as well as against the dermatophytic fungus *Trichophyton rubrum* (MIC = 6.7 μM). Importantly, the peptide displayed no hemolytic activity and no detectable toxicity toward mammalian cell lines *in vitro*. *Cm*-p5 itself was derived from *Cm*-p1 (SRSEL­IVHQR), a peptide originally identified in the marine snail *Cenchritis muricatus* by the same research group.^124,125^ These cyclic derivatives showed improved structural stability, particularly through stabilization of the α-helical conformation. The cyclic monomer CysCys-*Cm*-p5 (*i+4*) displayed enhanced antifungal activity against *Candida albicans* (MIC = 3.5 μM) and *Candida parapsilosis* (MIC = 9.8 μM), whereas dimer 2 (antiparallel) unexpectedly exhibited antibacterial activity against *Listeria monocytogenes* (MIC = 4.4 μM). In addition, neither the cyclic monomer nor the dimeric peptides showed detectable toxicity. The combination of antimicrobial activity, low cytotoxicity, and α-helix stabilization suggests improved metabolic stability and potential *in vivo* activity for these derivatives.^123^

In addition to cyclization, PEGylation, the covalent attachment of polyethylene glycol (PEG) chains to peptides, has been employed to improve the pharmacokinetic properties of AMPs, including solubility, serum stability, and reduced toxicity. A representative example is the PEGylation of the antimicrobial peptide LyeTxI-b (IWLT­ALKF­LGKN­LGKL­AKQQ­LAKL-NH_2_; MICs = 1.0–2.0 μM against Gram-negative and Gram-positive bacteria), developed by researchers from Brazil. This peptide is derived from a venom peptide originally isolated from the Brazilian spider *Lycosa erythrognatha*. The PEGylated derivative was obtained from the analog LyeTxI-bCys (Ac-IWLT­ALKF­LGKN­LGKLA­KQQC­AKL-NH_2_; MICs = 1.0–2.0 μM), in which the leucine residue at position 21 was replaced by cysteine, enabling conjugation via Michael-type addition to a maleimide-functionalized PEG moiety. PEGylation preserved antimicrobial activity (MICs of 4.0–8.0 μM against Gram-negative and Gram-positive bacteria) while significantly reducing cytotoxicity and hemolytic effects, enhancing resistance to proteolytic degradation, and modulating peptide-membrane interactions.^126^ In earlier work, the same group demonstrated that PEGylated LyeTxI-b exhibited strong activity against carbapenem-resistant *Acinetobacter baumannii* (CRAB) in an experimental pneumonia model, with an improved safety profile compared to the nonmodified peptide. Notably, LyeTxI-bPEG (Figure 1A), showed *in vivo* efficacy comparable to polymyxin treatment, whereas the non-PEGylated peptide displayed reduced activity, similar to the untreated control group. This difference is consistent with the known instability of LyeTxI-b, which is susceptible to degradation by plasma peptidases, leading to reduced antibacterial activity in the presence of human plasma and serum. In contrast, PEGylation protected the peptide from serum-mediated inactivation, thereby improving its biological stability. Additionally, after 21 days of resistance induction assays using CRAB, the MIC of LyeTxI-b increased 8-fold, while that of colistin increased 4-fold; in contrast, the MIC of LyeTxI-bPEG remained unchanged throughout the evaluation period. PEGylation also inhibited the development of resistance in the tested bacterial strain, indicating that this modification enhances the stability of the peptide against microbial resistance mechanisms. These findings underscore the relevance of PEGylation for advancing AMPs toward clinical application.^127^

In addition, a collaborative study between Brazil and Uruguay investigated the derivative LyeTx I mnΔK (IWLTK­ALKFL­GKN­LGK-NH_2_), a shortened version of LyeTx I containing a lysine residue at position 5, introduced to enhance both positive charge and amphiphilicity. LyeTx I mnΔK was radiolabeled with ^68^Ga via conjugation to a 1,4,7,10-tetraazacyclododecane-1,4,7,10-tetraacetic acid (DOTA) chelator at Lys-16, enabling the evaluation of its distribution in rat models of *Staphylococcus aureus* muscle infection. The authors further demonstrated that *In vitro* binding assays the radiopharmaceutical exhibits high affinity for *S. aureus* cells, with binding percentages increasing as a function of bacterial concentration. Biological data further showed that ^68^Ga-DOTA-LyeTx I mnΔK presents radiochemical stability above 95% and a favorable biodistribution profile, with predominant renal clearance. Imaging studies revealed that the compound clearly identifies muscle infection foci caused by viable *S. aureus*, displaying significantly higher uptake compared to sterile inflammatory tissues (induced by turpentine). This selectivity, combined with its low observed toxicity, highlights this spider venom-derived peptide as a promising candidate for the development of more precise diagnostic agents in humans.^78^

From a peptide engineering perspective, this shortened variant had been previously characterized by its cationic nature and its ability to adopt a helical conformation in membrane-mimicking environments. It was designed alongside two additional variants, the *N*-acetylated LyeTx I mnΔKAc and LyeTx I mn (IWLT­ALKFL­GKN­LGK-NH_2_), a 15-amino-acid sequence derived from residues 1–15 of the original peptide, to preserve biological activity while reducing toxicity and hemolysis. Among these variants, LyeTx I mnΔK retained excellent inhibitory activity, with a minimum inhibitory concentration (MIC) of 0.96 μM against *Escherichia coli*, outperforming the original peptide in this case. For other microorganisms, MIC values ranged from 1.09 μM against *Acinetobacter baumannii* to 5.6 μM against *Staphylococcus aureus* and 4.90 μM against the fungus *Cryptococcus neoformans*. In contrast, the lysine-deficient derivative (LyeTx I mn) was considerably less effective, underscoring the importance of positive charge in mediating interactions with bacterial membranes. Beyond *in vitro* assays, the study advanced to *in vivo* models, where LyeTx I mnΔK was evaluated in the treatment of *S. aureus*-induced septic arthritis in mice. The results were significant, showing a marked reduction in local bacterial burden and inflammation, assessed by neutrophil infiltration and cytokine levels, as well as a pronounced antinociceptive (analgesic) effect. Altogether, these findings indicate that LyeTx I mnΔK is a strong candidate for the development of new antimicrobial agents, as it combines a simplified structure with high efficacy and significantly reduced toxicity compared to the native spider peptide.^128^

In comparison, the incorporation of *D*-amino acids has been explored to a lesser extent in Latin American AMP research and is often applied more indirectly. Rather than being systematically integrated into peptide backbones, *D*-amino acids have frequently been investigated as modulators of the biofilm architecture and stability. For example, studies conducted in Brazil demonstrated that *D*-amino acids, in combination with antimicrobial peptides, such as LL-37, can significantly disrupt multispecies biofilms. These findings highlight their potential as adjuvants that enhance peptide efficacy, particularly in biofilm-associated infections, although their use as structural components of engineered AMPs remains limited in this region.^129^

Similarly, *N*-methylation, lipidation, and glycosylation are well-established strategies for enhancing peptide performance and remain underexplored in AMPs engineering in Latin America. Although widely recognized in peptide therapeutics, their application in the region is still limited, reflecting a broader trend in which only a subset of chemical modification strategies have been effectively implemented. Research efforts have therefore focused on approaches that provide a favorable balance among synthetic feasibility, structural stability, and biological performance, while other strategies, such as *D*-amino acid incorporation and these same modifications, remain promising but comparatively less developed.

Rational design may involve various modifications to their scaffold, such as the addition of fatty acid chains (lipidation) to increase membrane affinity and proteolytic stability, or the conjugation of sugars (glycosylation) to improve selectivity and antibiofilm activity.^130,131^ Building upon these modification strategies, the conjugation of antibiotics or drugs via spacers, which under specific conditions can trigger the release of the nonpeptide moiety, can enhance effects through synergism. A notable example of this approach in Brazil is the study exploring the series of cell-penetrating peptides AcYG­(RRLL)_n_-NH_2_, AcYG­(RLRL)_n_-NH_2_, AcYG­(RRWW)_n_-NH_2_ (where n = 1–4) and their *N*-terminal conjugation with linezolid and benznidazole. These conjugates were designed to enhance antibacterial and antiparasitic activities, respectively.^132^

Similarly, in a collaborative effort involving three different research groups in Colombia, the chemical conjugation of AMPs derived from buforin AGRG­KQGG­KVRA­KAKT­RSSR­AGLQ­FPVG­RVHR­LLRK­GNK; derivatives: RLLR and RLLRLLR) and lactoferricin B (FKCR­RWQW­RMKK­LGAP­SITCV­RRAF; derivatives: RRWQWR and RWQW­RWQWR) at the *N*-terminus with nonpeptide moieties, such as 6-aminohexanoic acid (Ahx), ferrocene (Fe), caffeic acid (CA), ferulic acid (FA), and oxolinic acid (OA), has proven effective in enhancing antimicrobial activity and modulating interactions with target membranes. For example, incorporating the conjugation motifs oxolinic acid and 6-aminohexanoic acid (OA-Ahx) into the AMP RRWQWR decreased the MIC by 2.5-fold against *E. coli* ATCC 25922 and by 5-fold against *S. aureus* ATCC 25923. A representative OA-Ahx peptide conjugate is shown in Figure 1A.^133^

In the context of combating neglected diseases, studies conducted in Brazil have investigated bioactive AMPs suck temporins-class peptide TSHa (FLSGIVGMLGKLF) and the nonmembranolytic dimeric cationic peptide p-Bt [(KKYR­YHLKPF)­2K-NH_2_], an analogue of the BthTX-I peptide from *Bothrops jararaca* venom, whose *N*-terminal conjugation with guanidine groups resulted in enhanced activity against *Leishmania* spp. while reducing cellular toxicity.^134^ This demonstrates that AMP conjugation is a promising strategy for the development and identification of new drugs for the treatment of bacterial infections.

In parallel with conjugation strategies, the design of hybrid and chimeric AMPs has emerged as a powerful strategy to enhance antimicrobial potency, broaden the spectrum of activity, improve physicochemical properties, reduce toxicity, and enhance stability. Hybrid peptides are generated by combining functional domains from different parental AMPs, whereas chimeric peptides involve the fusion of distinct peptide fragments to optimize structure–activity relationships. In Latin America, this approach has gained increasing attention, particularly in countries such as Brazil and Mexico, where advances in peptide chemistry and computational biology have supported the rational design of novel molecules.

Within this framework, researchers in Mexico have developed novel chimeric peptides derived from well-known AMPs, including pandinin-2 (FWGA­LAKG­ALKL­IPSLF­SSFS­KKD) from the scorpion *Pandinus imperator*, ascaphin-8 (GFKD­LLKG­AAKA­LVKT­VLF) from the skin of the frog *Ascaphus truei*, and maximin-3 (GIGGK­TLSG­LKTA­LKGA­AKELA­STYLH) from the skin secretions of the toad *Bombina maxima*. The resulting chimeras, chimera-1 (GFKD­LLKG­AAKA­LVKT­VLFF­SKKD), chimera-2 (GIGG­KTLS­GFKD­LLKG­AAKA­LVKT­VLF), and chimera-3 (GIGG­KTLS­GFKD­LLKG­AAKA­LVKT­VLFF­SKKD), were designed to combine key structural motifs from the parental peptides. Chimera-1 incorporates the *C*-terminal region of pandinin-2 into ascaphin-8, whereas chimera-2 includes the *N*-terminal region of maximin-3 fused to the *N*-terminus of ascaphin-8. Chimera-3 integrates both modifications, combining the *N*-terminal region of maximin-3 and the *C*-terminal region of pandinin-2 with ascaphin-8. These designs resulted in enhanced antimicrobial activity against clinically relevant bacterial strains without clear selectivity between Gram-positive and Gram-negative bacteria.^135^

In Brazil, hybrid peptide engineering has increasingly relied on interdisciplinary strategies integrating peptide synthesis, structural biology, and computational modeling. A recent study exemplifies this approach by designing chimeric peptides derived from cecropin and cathepsin sequences. The process involved multiple sequence alignment of cecropin variants (A, B, C, and D) and different classes of cathepsins (B, K, L, S, E, and F) to identify conserved structural motifs, which were recombined in distinct orientations at the *N*- and *C*-termini using bioinformatics tools and machine learning algorithms. The resulting candidates were then subjected to rigorous in silico screening, including evaluation of physicochemical properties, cell-penetrating potential, and antimicrobial activity, while preserving the amphiphilic character required for membrane interaction and leveraging the structural stability of cathepsins. The precursor sequences underlying the most promising chimeras were derived from conserved motifs of cecropin B and cathepsin L. The synthesized and tested peptides included CATL1.2_CECB2 (YWLV­KNSW­GKIFK­KIE), CATL1.2_CECB1_2 (WLVK­NSWGK­LGKKI), and CECB1_CATL1.2 (KIGK­KIEY­WLVK­NSWG). Biological assays revealed robust activity, particularly against Gram-negative bacteria and yeasts, with MIC values ranging from 67.3 to 307.7 μM against *Escherichia coli* (ATCC 25922), *Staphylococcus aureus* (ATCC 25923), and *Candida albicans* (ATCC 10231). Biofilm inhibition assays were performed with the most active peptides, highlighting CATL1.2_CECB2 for efficient bacterial killing, while CECB1_CATL1.2 showed strong efficacy in eradicating *E. coli* at 5× and 10× MIC. The safety profile of these compounds was supported by low hemolytic activity in human erythrocytes (below 5%) and the absence of cytotoxicity in endothelial cell lines (HUVEC) at concentrations up to 265 μM. Furthermore, *in vivo* assays using the *Galleria mellonella* larval model demonstrated no systemic toxicity, reinforcing their therapeutic potential. Mechanistic insights, supported by molecular docking studies, suggest that these chimeras act not only through membrane disruption but also via interactions with critical intracellular targets, such as bacterial DNA gyrase and thymidylate synthase.^136^

Similarly, in Colombia, notable advances have been achieved in the design of chimeric AMPs combining motifs from bovine lactoferricin B (LfcinB) and buforin II (BFII). These chimeras, developed for the treatment of cryptococcosis, were constructed by fusing the fragments LfcinB_20–25_ (RRWQWR) and BFII_32–35_ (RLLR) through a non-natural linker, 6-aminohexanoic acid (Ahx), to preserve structural flexibility and optimize interactions with the biological target. Among the resulting peptides, C6 (KKWQWK-Ahx-RLLRRLLR) showed the highest activity, with a MIC of 6.25 μg·mL^–1^ (3 μM) against most strains of *C. neoformans* var. *grubii*, while C5 (RRWQWR-Ahx-KLLKKLLK) exhibited a MIC of 12.5 μg·mL^–1^ (6 μM); in contrast, the parental peptides displayed negligible activity. Both chimeras demonstrated rapid fungicidal effects at near-MIC concentrations and strong synergism with fluconazole, enabling dose reduction of the conventional drug. Additionally, low cytotoxicity was observed in human fibroblasts, with cell viability remaining above 65% at effective concentrations. Overall, these findings highlight that the rational combination of functional motifs can significantly enhance antifungal activity and therapeutic potential.^137^

Beyond these country-specific efforts, the region has increasingly adopted computational and artificial intelligence approaches to the AMP design. Recent perspectives highlight how machine learning and multiobjective optimization are being used to design peptides with improved antimicrobial activity while minimizing toxicity, representing a shift toward data-driven peptide engineering.^138^ Overall, hybrid and chimeric peptide design in Latin America reflects a transition from the study of naturally occurring AMPs to rational and computationally guided engineering strategies. Although still developing compared with leading global research centers, the region shows strong potential due to its biodiversity, growing scientific infrastructure, and increasing integration of advanced design methodologies.

In recent years, regional initiatives have increasingly combined computational and experimental approaches into integrated AMP design pipelines. Artificial intelligence and machine learning models, including deep learning architectures that can generate and rank candidate sequences, are now employed alongside molecular modeling, docking simulations, and SAR analyses to refine physicochemical parameters such as net charge, hydrophobicity, amphipathicity, and secondary structure propensity.^16,139^ These *in silico* methodologies enable efficient exploration of vast peptide sequence spaces and support the rational prioritization of candidates with improved antimicrobial activity and reduced toxicity. In contrast to structural modification strategies, which focus on introducing specific changes into peptide scaffolds, computational approaches provide a framework for predicting, optimizing, and selecting peptide sequences prior to experimental validation. Although several foundational computational strategies were initially developed outside the region, research groups in Latin America have progressively incorporated these tools into their AMP development workflows. This integration has contributed to a transition from predominantly empirical and descriptive approaches to more data-driven and hypothesis-oriented peptide engineering. Together, the integration of bioinformatics, molecular modeling, and artificial intelligence is reshaping AMP discovery workflows, enabling more efficient candidate prioritization and accelerating the development of peptides with improved biological and pharmaceutical properties.^16,133,139^ These integrated strategies and iterative optimization processes can be conceptualized as a translational pipeline linking natural peptide discovery to the development of clinically relevant candidates while highlighting key physicochemical and biological challenges that must be addressed (Figure 3).

**Figure 3:**
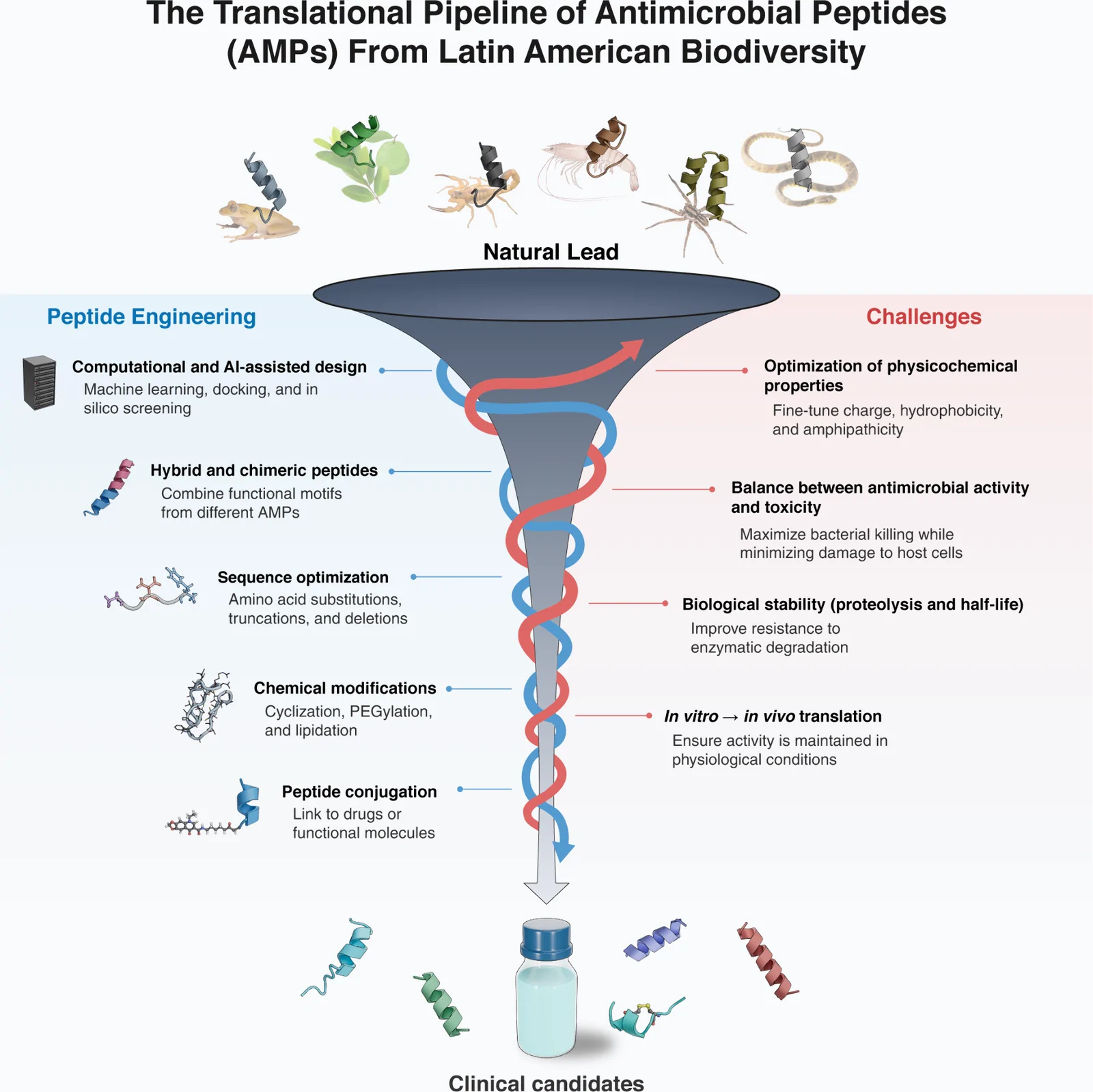
Conceptual framework of the translational pipeline of antimicrobial peptides (AMPs) derived from Latin American biodiversity. The schematic illustrates the progression from natural leads through iterative and multidisciplinary engineering strategies aimed at improving antimicrobial performance and therapeutic potential. These strategies include sequence optimization, chemical modification, peptide conjugation, and hybrid or chimeric design, often supported by computational and artificial intelligence (AI)-assisted approaches for the rational refinement of physicochemical and structural properties. In parallel, the figure highlights key translational challenges that constrain AMP development, including the optimization of charge, hydrophobicity, and amphipathicity, the balance between antimicrobial efficacy and host-cell toxicity, the improvement of resistance to proteolytic degradation, and the transition from *in vitro* activity to *in vivo* efficacy. Collectively, this framework emphasizes the central role of rational design and integrated engineering strategies in advancing natural peptide scaffolds toward clinically relevant candidates.

Another approach to AMP development involves the integrating computational modeling, SAR analyses, and sequence engineering strategies to generate molecules with enhanced antimicrobial activity and reduced toxicity. Within this framework, a representative example is provided by AMPs derived from hylin-Pul3 (FLGA­LIPAI­AGAIG­GLIRK-NH_2_), a peptide isolated from the South American frog *Boana pulchella*. Conducted through a collaboration between Argentine and Brazilian groups, this research addressed the parent peptide’s significant hemolytic activity and its MICs of 14 μM and 108 μM against *S. aureus* and *E. coli*, respectively, by designing new derivatives with a net charge greater than +2, α-helical-forming sequences, and specific synthetic constraints. To support this rational design process, characterization and evaluation were performed using bioinformatics analyses with established online tools, including ProtParam, GRAVY, HNN, HeliQuest v1.2, AlphaFold2, CAMPR3, the DBAASP v3 platform, HAPPENN, and HemoPred. These approaches enabled the optimization of key physicochemical properties, such as sequence length reduction, modulation of hydrophobicity, increased cationicity, charge distribution, and the design of amphipathic structures associated with potent antimicrobial activity and low cytotoxicity. As result, six candidate peptides were identified: dHP3-31 (LWPA­IRGA­IKKL­IKK-NH_2_), dHP3-50 (RLGA­LIPAI­RGAI­KKLIKK-NH_2_), dHP3-50.137 (RAGA­LWPA­IRGA­IKKI­IKK-NH_2_), dHP3-50.190 (RRIA­LIPA­IRGA­IKKL­IKK-NH_2_), dHP3-84 (RLGK­LIPR­IAKA­IGRL­IRK), and dHP3-84.39 (RRGK­LIPRL­AKAI­GRLI­RK). These derivatives exhibited enhanced activity against Gram-negative bacteria and improved selectivity in hemolytic assays, particularly for those with imperfect amphipathicity. Finally, in fibroblast assays, dHP3-84 was well-tolerated, while dHP3-84.39 promoted cell proliferation. In addition, selected derivatives exhibited complementary biological activities beyond antibacterial effects, including antiviral activity against *Varicellovirus suidalpha1* and measurable antioxidant properties. These findings suggest that hylin-derived peptides can be successfully engineered to combine antimicrobial activity with additional biological properties, including antiviral and antioxidant effects. This example illustrates how computationally guided sequence optimization can diversify peptide functionality while preserving anti-infective efficacy.^140^

The Franco’s Lab has employed computer-aided automated design using the Joker algorithm, a tool that leverages AMP databases to search for patterns, such as template sequences and amino acid motifs, to generate novel or improved peptide variants with antimicrobial properties.^141^ Within this context, notable examples include the design of the peptides PaDBS1R2 (PMKL­LKRL­GKKI­RLAA­AFK), R3 (PMAK­LLPR­IKKK­ILAA­AFK), and R7 (PMAR­NKPK­ILKR­ILAK­IFK). These are characterized by the presence of Gly and Pro residues, which induce backbone flexibility; from these residues, key motifs can be derived that, when inserted, enhance killing performance against microorganisms. In assays against Gram-positive and Gram-negative bacteria, all peptides demonstrated activity except against *Enterobacter cloacae* (1383251) and carbapenemase-producing *Klebsiella pneumoniae* (KpC+001825971). Among them, PaDBS1R3 exhibited the most limited antibacterial spectrum, inhibiting only 6 of the 11 strains tested. Conversely, PaDBS1R2 and PaDBS1R7 showed the most potent activities, with MICs ranging from 2 to 8 μM against susceptible bacteria such as *Escherichia coli* and *Klebsiella pneumoniae*, as well as clinical and multidrug-resistant (MDR) isolates of *Acinetobacter baumannii*, *E. coli*, and *P. aeruginosa*. Both were also capable of inhibiting MRSA (Methicillin-resistant *Staphylococcus aureus*) with an MIC of 16 μM. Mechanistically, atomic force microscopy (AFM) studies indicated that these peptides could destabilize the bacterial membrane, causing increased surface roughness and loss of cellular integrity, particularly in *E. coli* and *P. aeruginosa* at higher concentrations. Beyond their antimicrobial activity, the peptides showed low toxicity toward mammalian cells, displaying no hemolytic effects or cytotoxicity across various human and murine cell lines up to 100 μM. Interestingly, PaDBS1R7 also demonstrated cytotoxic activity against cancer cells (IC_50_ ∼ 50 μM), suggesting a potential dual therapeutic role.^142^

Similarly, another approach explored by our group and collaborators is computer-aided design, which identifies patterns and motifs in AMP sequences that confer antimicrobial activity. Recent and ongoing initiatives include the evaluation of peptides engineered for specific therapeutic applications, such as the FK18 peptide (FCKL­FKRV­YRFY­KHW­AHK), which exhibited antibacterial activity targeted at the treatment of mastitis in livestock. This study demonstrated how sequences optimized through bioinformatics tools, specifically the Antimicrobial Peptide Database 3 (APD3) software, can be effectively applied in clinical and veterinary contexts with a focus on efficacy and safety. Regarding its potency, the FK18 peptide showed MIC values of 5 μM for *Staphylococcus aureus* isolated from bovine milk and 10 μM for *Escherichia coli* isolated from ovine milk.^143^ Another study conducted by researchers from Franco’s Lab in collaboration with international institutions reported the design of the AMP guavanin 2 (RQYM­RQIE­QALR­YGYR­ISRR) through *in silico* optimization of the guava (*Psidium guajava*) peptide Pg-AMP1 (RESP­SSRM­ECYE­QAER­YGYG­GYGG­GRYG­GGYG­SGRG­QPVG­QGVE­RSHD­DNRN­QPR) using a genetic algorithm. Guavanin 2 adopts an α-helical structure and exhibits potent activity mainly against Gram-negative bacteria, including *Escherichia coli* and *Acinetobacter baumannii*, with MIC values of 6.25 μM. It also shows activity against *Pseudomonas aeruginosa* (25 μM) and *Klebsiella pneumoniae* (80 μM), while displaying weaker activity against Gram-positive bacteria (50–100 μM). The peptide exhibits limited antifungal activity and is inactive against *Candida albicans*, but shows low cytotoxicity toward human cells at concentrations above 200 μM. This study highlights the potential of computational design strategies in Latin America to generate optimized AMPs with improved antimicrobial profiles.^144^

Building upon these advances, the use of deep learning and artificial intelligence to generate and optimize AMP sequences was recently demonstrated in a Colombian study, in which 26 *in silico*-generated peptides exhibited a high likelihood of antimicrobial activity and were experimentally validated against bacterial and fungal pathogens. Nine of these peptides showed MIC values below 10 μM, with some achieving inhibitory concentrations as low as 2 μM, such as OrP1M (LAKR­WLKLL­GKLAK) against *Escherichia coli*, OrP9M (IGKV­WKVA­KKLIKI) against *Pseudomonas aeruginosa*, and VeP1 (FKKL­LKFLK­WLF) against *Staphylococcus aureus*. The sequences were generated using PepGen 1.0 (a synthetic peptide generator trained with Recurrent Neural Networks (RNN) and Long Short-Term Memory (LSTM) cells) and AmPepGen (an antimicrobial peptide generator trained with Generative Adversarial Networks (GAN)). Candidates were selected and optimized based on predicted antimicrobial activity probabilities greater than 95%, according to the classification models CAMPR3, AMP Scanner v2, and AmpClass 1.0. Furthermore, biosecurity assessments for toxicity and hemolytic activity were predicted using ToxinPred and HAPPENN, respectively.^145^ Furthermore, *in silico* analyses of peptides derived from medicinal plants illustrate how bioinformatics can characterize and predict AMP properties prior to experimental validation.^146^

More broadly, in recent years, approaches based on genetic algorithms, machine learning, and artificial intelligence have been proposed to efficiently explore the vast peptide sequence space, balancing parameters such as hydrophobicity, net charge, length, secondary structure, and cellular selectivity. These include natural language processing (NLP) models applied to biological sequences and deep learning strategies that enable the identification of complex patterns and the *in silico* generation of promising new candidates, accelerating the discovery and prioritization of peptides with a high probability of antimicrobial activity. In parallel, these optimization and classification tools enable the screening of large virtual peptide libraries, reducing experimental costs and accelerating the transition from empirical discovery to rational engineering with potential clinical application.^16,139^ Latin American regions, recent works demonstrate the application of these approaches: the *in silico* prospection of AMPs in Amazonian soil metagenomes using machine learning methods identified 692 candidate sequences using the AMPEPy tool, which utilizes physicochemical properties, structural features, and similarity patterns to classify peptides with antimicrobial potential.^23^ In another case, the development of machine learning-based tools to identify and characterize AMPs in genomic and transcriptomic data, such as AMP-Identifier, that relies on machine learning models (SVM, KNN, Random Forest, Decision Tree, Naive Bayes, and Neural Networks) trained on data sets prelabeled with physicochemical data, was enabled the identification of five new defensins (RcDef1–5) in the *Ricinus communis* genome. These were characterized through bioinformatics tools, physicochemical analysis, and molecular modeling.^147^

Similarly, the TACaPe tool, which employs a transformer-based model, was used to identify 100 potential antibacterial AMPs. The study, conducted in Brazil, focused on *P. aeruginosa* and used molecular docking against the quorum-sensing receptors LasR, RhlR, and PqsR to select candidates. Five of these candidates were synthesized, and *in vitro* assays against the ATCC 27853 strain revealed strong antibiofilm activity, with inhibition ranging from 23% to 93.4%, despite relatively high MIC values (125 to 250 μg·mL^–1^). Notably, in synergy assays with Meropenem, two peptides stood out, showing enhanced activity with combined MIC values of 31.25/0.02 and 62.5/0.04 μg·mL^–1^ for TAC-p1 (RRWF­RWWRV) and TAC-p4 (AKWR­VKWW), respectively. Cytotoxicity and hemolytic activity assays indicated that the AMPs are potentially safe at the concentrations required for antibacterial activity. Overall, these results reinforce the utility of computational tools in the discovery and optimization of new antimicrobial candidates, highlighting their potential as promising alternatives for the treatment of infections caused by multidrug-resistant (MDR) *P. aeruginosa*.^148^

A growing approach is biotechnological research focused on *in silico* prospecting, such as the identification of new AMPs derived from plants of the genus *Cereus*, popularly known as mandacaru. For example, the study on three Neotropical species, *Cereus fernambucensis*, *Cereus hildmannianus*, and *Cereus jamacaru*, all found in Brazil, where AMPs were identified through a computational pipeline that analyzed RNA-Seq (transcriptomic) data from the plants, performed BLASTp searches against global AMP databases, and predicted structure and toxicity using AlphaFold2. As a result, 13 promising sequences were identified, belonging to three main classes such as snakins, defensins, and chitinases. Among the highlighted AMPs, the peptide CF267 (snakin), found in *C. fernambucensis* and *C. jamacaru*, proved to be one of the most promising, with its partial sequence containing five disulfide bonds that confer structural stability to the molecule. As this is a computational study, predictions from molecular docking and molecular dynamics simulations indicated that some AMPs interact with critical bacterial targets, such as bacterial RNA polymerase, suggesting a high inhibitory potential against clinically relevant pathogens. These include Gram-positive bacteria such as *Staphylococcus aureus* and *Streptococcus epidermidis* as well as Gram-negative bacteria such as *Pseudomonas aeruginosa* and *Escherichia coli*. Simulations demonstrated that the peptide CF267 maintains a stable structure under simulated physiological conditions, highlighting it as a strong candidate for future chemical synthesis and experimental validation against these pathogens.^149^

Recent studies highlight the biotechnological potential of proteins and peptides from *Chenopodium quinoa* and related Andean pseudocereals. Beyond their nutritional value, quinoa contains bioactive compounds such as lunasin, a 43-amino-acid peptide with antioxidant, anti-inflammatory, and anticancer properties, and quinoine, a type 1 ribosome-inactivating protein active against glioblastoma. Additionally, peptides generated by *in vitro* digestion of quinoa proteins have shown inhibitory effects on colon cancer cell proliferation.^150^ Similarly, *Amaranthus* species, including *Amaranthus hypochondriacus* L. cv. Criolla, contain lunasin-like peptides with anticancer activity, reinforcing the potential of these pseudocereals as sources of functional biomolecules.^151^ In this context, recent efforts have increasingly focused on the anti-infective potential of quinoa-derived peptides, with a study by researchers from Ecuador, Chile, and Brazil reporting the development of new AMPs derived from chenopodin, the main storage protein of quinoa seeds (*Chenopodium quinoa*), using an *in silico* mining approach. Regions with antimicrobial potential were identified using tools such as AMPA, AMPfun, and AMP Scanner, resulting in the fragments Chen1 (YGVR­GRGRIQ­IVNA) and Chen2 (AHSII­YGVR­GRGRI). Based on the Chen1 peptide, synthetic variants were generated through rational design, in which asparagine and glutamine residues were replaced with amino acids known to enhance antimicrobial activity such as arginine, to increase positive charge (ChenR, YGVR­GRGR­IRIVRA), and tryptophan, to increase hydrophobicity (ChenW, YGVR­GRGR­IWIV­WA). The results showed that, while Chen1 was inactive (>512 μM), the modified variants exhibited high potency, with ChenW being the most effective, showing a MIC of 8 μM against both bacteria, followed by ChenR, which displayed greater selectivity toward *E. coli* (MIC of 16 μM) compared to *S. aureus* (128 μM). In contrast, Chen2 displayed moderate activity, with MIC values of 64 μM against *E. coli* and 128 μM against *S. aureus*. Mechanistic studies indicated that these peptides act primarily through interactions with the cell membrane, leading to its destabilization and loss of integrity. Membrane permeabilization assays using propidium iodide, together with computational simulations, suggest pore formation as the underlying mechanism, which is characteristic of AMPs. In addition to antibacterial activity, the peptides also demonstrated antiviral potential against SARS-CoV-2. Chen2 and ChenR exhibited EC_50_ values in the nanomolar range (407.8 nM and 345.3 nM, respectively), along with low cytotoxicity, indicating a promising therapeutic window.^152^

## 4 Translational and Biotechnological Challenges for AMP Development in Latin America in Anti-infective Applications

While the field of AMPs has advanced globally, the systematic integration of data derived from Latin American biodiversity remains limited. Global reviews and patent landscape analyses reveal that, although the Neotropical region hosts the highest diversity of amphibians, one of the primary natural reservoirs of AMPs, it accounts for only ∼ 3.7% of granted patents on peptides derived from these animals. In contrast, Asia and North America account for 65.4% and 15.4% of patents, respectively, highlighting a significant underutilization of local biodiversity in biotechnology.^28^ Despite harboring one of the world’s greatest biological diversities, which represents a vast source of bioactive peptides, as demonstrated by identification studies, much of the knowledge regarding AMP modifications generated in Latin America in recent years remains scattered across regional studies, theses, dissertations, and institutional technical reports. These works frequently focus on describing specific molecules or on isolated biological assessments, lacking comprehensive comparative analyses linking structural diversity, biophysical properties, and antimicrobial activity. This fragmentation hinders the consolidation of knowledge and limits the application of modern rational design approaches at the regional scale, as further evidenced by analyses of the innovation and patent landscapes. Even on a global scale, the clinical translation of AMPs faces significant barriers, cytotoxicity. These factors contribute to the still-limited adoption of these compounds compared to conventional antibiotics.^27^

The translation of AMPs into clinically viable anti-infective agents is hindered by multiple scientific and technological limitations. In Latin America, these challenges are further exacerbated by structural constraints, including limited funding for research and innovation, insufficient regulatory frameworks, and gaps in surveillance and implementation capacity, which collectively restrict the development and translation of novel antimicrobial strategies.^153–155^ Although these compounds have demonstrated high potential against multidrug-resistant pathogens, their adoption as therapeutic, veterinary, and industrial solutions remains limited by technological bottlenecks, regulatory hurdles, and financial constraints that disproportionately affect developing countries. Recent state-of-the-art reviews on AMP development indicate that key limitations include susceptibility to proteolytic degradation, dose-dependent cytotoxicity, poor pharmacokinetic profiles, and low bioavailability, as well as challenges associated with large-scale production and delivery systems, all of which continue to hinder their clinical translation.^156–159^ From a regulatory and technological standpoint, AMPs face additional hurdles, as short peptides (typically <40 amino acids) do not fall under a dedicated regulatory category and are instead evaluated within existing small-molecule or biologics frameworks, which may not fully capture their unique properties. Even when derived from biological sources, their synthetic nature means that, under FDA interpretation, they are not classified as biological products; instead, they are regulated as small-molecule drugs, which significantly affect their licensing and approval pathways.^160^ This goes hand in hand with the fact that in many Latin American countries, regulatory agencies still rely on FDA-based guidelines for small-molecule drugs. Since these are obtained through chemical synthesis, they require extensive toxicological, pharmacokinetic, and stability studies, which demand an advanced infrastructure and substantial investment. Technological strategies aimed at improving the pharmacological profile of AMPs, such as nanoparticle encapsulation, chemical conjugation, and topical formulations, are often necessary; however, these approaches also increase the regulatory complexity and development costs.^27^

These technological challenges are closely related to financial bottlenecks. Market analyses and prospective studies indicate that AMP development entails initial costs significantly higher than those of conventional antibiotics, primarily because of specialized synthesis, purification, formulation, and preclinical testing requirements. In particular, antimicrobial peptides require complex manufacturing processes and high-cost production pipelines, with peptide synthesis and purification representing major economic barriers to large-scale implementation and commercialization.^161^ This scenario makes fundraising more difficult and increases the perceived risk for investors, hindering the progress of promising candidates beyond the early stages of the research. This limitation is particularly critical in Latin America, where funding for biomedical innovation is more constrained.

The limited resources available for bioprospecting and translational research represent another significant hurdle. Despite Latin America’s vast biodiversity, public investment remains largely concentrated in basic research, with insufficient sustained funding for the intermediate and advanced stages of drug development. As a result, many AMPs remain confined to academic settings and fail to progress toward commercialization or clinical evaluation.^162^

## 5 Conclusions and Future Outlook

Although the development of antimicrobial peptides in Latin America faces important scientific, technological, regulatory, and financial challenges, the region also presents clear strategic opportunities for innovation. One of the most important needs is the implementation of coordinated strategies to integrate the growing volume of knowledge generated across the regional research groups. The development of integrated peptide databases and the broader adoption of computational approaches, including machine learning and multiobjective optimization, could significantly expand the diversity and quality of AMP candidates, accelerating their evolution from natural molecules into viable therapeutic leads.

Another key step involves strengthening the collaborative networks across the region. Partnerships involving universities, research institutes, governmental agencies, and industry can facilitate technology transfer, shared infrastructure, and capacity building in essential areas, such as computational modeling, preclinical studies, and regulatory development. Moreover, participation in international consortia addressing antimicrobial resistance enables Latin American research to integrate with global initiatives and expand the visibility and impact of its findings. An example of this type of collaboration is the joint effort between the Pan American Health Organization and the Global Antibiotic Research & Development Partnership, which aims to strengthen antibiotic research, development, and access in Latin America and the Caribbean, highlighting the importance of coordinated international action against antimicrobial resistance.^163^ More recently, PAHO has further reinforced this regional strategy through the launch of a clinical trial accelerator designed to enhance research capacity, streamline multicenter studies, and promote faster and more efficient development of health technologies across the Americas.This initiative underscores the growing commitment to building robust collaborative research ecosystems capable of responding to urgent public health challenges, including antimicrobial resistance.^164^

Furthermore, incorporating AMPs into one health strategies, which integrate human, animal, and environmental health, can strengthen the alignment of these strategies with regional public policies. Recent analyses of antimicrobial stewardship programs in Latin America indicate growing recognition of the need for alternatives to classical antibiotics, creating a favorable environment for adopting AMPs as part of integrated infection-control strategies.^165^ In summary, the combination of unique biodiversity, expanding scientific expertise, and increasing international collaboration positions Latin America as a potentially important contributor to the development of next-generation anti-infective agents. Overcoming existing bottlenecks will require integrated policies, targeted investments, and multisectoral cooperation, but it represents a promising path toward consolidating AMPs as relevant solutions to combat infectious diseases in the region.

## Notes

**Artificial Intelligence Assistance.** The English grammar and language of the manuscript were revised with the assistance of ChatGPT (OpenAI, free version). The revised text was subsequently reviewed and edited by a technical English language specialist. Throughout the revision process, all language changes were verified by human review before being incorporated into the manuscript. The authors reviewed and approved the final version and assume full responsibility for the scientific content, interpretation of the results, and conclusions presented in this manuscript.
